# Formation of HERV-K and HERV-Fc1 Envelope Family Members is Suppressed on Transcriptional and Translational Level

**DOI:** 10.3390/ijms21217855

**Published:** 2020-10-23

**Authors:** Victoria Gröger, Lisa Wieland, Marcel Naumann, Ann-Christin Meinecke, Beate Meinhardt, Steffen Rossner, Christian Ihling, Alexander Emmer, Martin S. Staege, Holger Cynis

**Affiliations:** 1 Department of Drug Design and Target Validation, Fraunhofer Institute for Cell Therapy and Immunology, Weinbergweg 22, 06120 Halle, Germany; victoria.groeger@izi.fraunhofer.de (V.G.); marcel.naumann@izi.fraunhofer.de (M.N.); ann-christin.meinecke@web.de (A.-C.M.); 2 Department of Neurology, Medical Faculty, Martin Luther University Halle-Wittenberg, Ernst-Grube-Str. 40, 06097 Halle, Germany; lisa.wieland@uk-halle.de (L.W.); beate.meinhardt@uk-halle.de (B.M.); alexander.emmer@uk-halle.de (A.E.); 3 Department of Surgical and Conservative Pediatrics and Adolescent Medicine, Medical Faculty, Martin Luther University Halle-Wittenberg, Ernst-Grube-Str. 40, 06097 Halle, Germany; 4 Paul Flechsig Institute for Brain Research, Leipzig University, Liebigstraße 19, 04103 Leipzig, Germany; Steffen.Rossner@medizin.uni-leipzig.de; 5 Department of Pharmaceutical Chemistry & Bioanalytics, Institute of Pharmacy, Martin Luther University Halle-Wittenberg, Charles Tanford Protein Center, Kurt-Mothes-Str. 3a, 06120 Halle, Germany; christian.ihling@pharmazie.uni-halle.de

**Keywords:** human endogenous retroviruses, expression, codon usage, transcription, translation

## Abstract

The human genome comprises 8% sequences of retroviral origin, so-called human endogenous retroviruses (HERVs). Most of these proviral sequences are defective, but some possess open reading frames. They can lead to the formation of viral transcripts, when activated by intrinsic and extrinsic factors. HERVs are thought to play a pathological role in inflammatory diseases and cancer. Since the consequences of activated proviral sequences in the human body are largely unexplored, selected envelope proteins of human endogenous retroviruses associated with inflammatory diseases, namely HERV-K18, HERV-K113, and HERV-Fc1, were investigated in the present study. A formation of glycosylated envelope proteins was demonstrated in different mammalian cell lines. Nevertheless, protein maturation seemed to be incomplete as no transport to the plasma membrane was observed. Instead, the proteins remained in the ER where they induced the expression of genes involved in unfolded protein response, such as *HSPA5* and *sXBP1*. Furthermore, low expression levels of native envelope proteins were increased by codon optimization. Cell-free expression systems showed that both the transcriptional and translational level is affected. By generating different codon-optimized variants of HERV-K113 envelope, the influence of single rare t-RNA pools in certain cell lines was demonstrated. The mRNA secondary structure also appears to play an important role in the translation of the tested viral envelope proteins. In summary, the formation of certain HERV proteins is basically possible. However, their complete maturation and thus full biologic activity seems to depend on additional factors that might be disease-specific and await elucidation in the future.

## 1. Introduction

In the course of evolution, a large number of retroviral elements were incorporated into the genomes of vertebrates. At present, the human genome consists of up to 8% of such transposable elements (TE) resulting from infections with ancient retroviruses, which circulated millions of years ago [1]. Since the integration occurred by infection of the germ line, the resulting provirus of the so-called endogenous retroviruses (ERV) can be transmitted vertically as a host allele [2,3]. The retroviral provirus typically possesses three coding regions: the group-specific antigen (*GAG*) gene, which provides the capsid- and matrix proteins of the nucleus, the polymerase (*POL*) gene encoding the viral enzymes for reverse transcription and integration into the host genome, and the envelope (*ENV*) gene, encoding the viral glycoproteins on the virus surface important for fusion with the host cell membrane during infection. Additionally, these regions are flanked on either side by long terminal repeats (LTR) that regulate transcription of enclosed viral genes [4].

Due to their long persistence in the genome, the proviruses were subject to selective pressure, which resulted in inactivation of most viral sequences by (I) disruption of open reading frames (ORFs) by mutations or (II) complete loss of all viral genes by recombination between flanking LTR sequences. As a consequence, nearly all human ERV (HERV) show defective protein products [5]. Nevertheless, there are few HERV with almost complete ORFs, which are able to form functional proteins. Under normal conditions proviral sequences are epigenetically silenced, but disease or developmental stages can activate transcription. As popular examples, the envelope proteins (*ENV*) of the HERV-W family, e.g., syncytin-1 and the HERV-FRD family, e.g., syncytin-2, are important during pregnancy and embryogenesis [6,7].

Besides a beneficial effect, some HERV ENV are thought to contribute to diseases including cancer and autoimmune disorders [8,9,10,11,12]. Transcriptional activation of retroviral elements in pathologic tissues of soft tissue sarcoma patients or elevated antibody titers against viral proteins in the sera of patients with autoimmune rheumatic diseases were observed [13,14,15]. Little is known about the protein expression of ENV in mammals. For understanding the biological role and the pathogenetic risk of ENV, a functional characterization including analysis of maturation, localization and limitations of native ENV expression is necessary. For this purpose, we investigated in this study the expression of three selected ENV possessing intact ORFs in the human genome and a known correlation to diseases, such as Multiple Sclerosis [16,17,18,19].

We focused on two members of ENV from the HERV-K family: HERV-K18 ENV and HERV-K113 ENV. Additionally, one member from the HERV-H/F family, HERV-Fc1 ENV, was investigated. HERV-K ENV belong to the human endogenous mouse mammary tumor virus-like (HML2)-subset of the HERV-K family, which comprises the most complete and biological active members among all HERVs [20,21,22]. The studied HERV-K18 on chromosome 1 is a locus of 9235 bp with three allelic variants. The variants K18.1 and K18.2 are most common in the Caucasian population (≈90%), but only K18.2 encodes a full-length ENV of 553 amino acids [23,24,25]. Interestingly, the K18.2 allele (NCBI accession no.: AF333069.1) is located in the first intron of the *CD48* gene, which can be transactivated by Epstein–Barr virus (EBV) and might promote the expression of ENV with putative super-antigenic properties [18,25,26,27]. The third allelic variant, K18.3, has been identified as risk factor for Multiple Sclerosis [28].

HERV-K113 is located on chromosome 19p12 (NCBI accession no.: NC_022518.1). Despite its short integration time, it possesses mutations in the reverse transcriptase gene, which leads to the loss of replication competency of the provirus [29]. Interestingly, the fixation of HERV-K113 in the human population is still ongoing. Today the provirus is present in about 30% of humans and it is one of few retroviruses with known ORFs for all viral proteins [30]. In addition, we studied HERV-Fc1 ENV, which is located on the X chromosome (NCBI accession no.: XM_011531085.2). HERV-Fc1 contains a full-length *ENV* open reading frame. In addition to small in-frame deletions and insertions, the *GAG* and *POL* regions contain stop codons. The stop codon in *GAG* is located near the 3′ end [31]. *POL* contains two stop codons and frame shifts. Compared with HERV-H consensus sequences [32] translation of this sequence would result in a protein with a large C terminal truncation. The HERV-Fc1 locus seems to be genetically associated with Multiple Sclerosis (MS) [19,33,34]. Unlike the HERV-K members, the HERV-Fc family has a very limited expansion with only a few proviruses in the human genome [35].

In the present study, we analyzed the expression of all three *ENV* in several mammalian cell lines with a closer view on protein localization and protein maturation. Our second aim was to examine reasons for the observed limited protein synthesis efficacy of native ENV. Therefore, we investigated rare codon bias as a factor for the limited protein biosynthesis of the native proteins of HERV-K113 in mammalian cell lines and in a cell-free system.

## 2. Results

### 2.1. ORFs of HERV-K18, HERV-K113, and HERV-Fc1 form Envelope Proteins

**Figure 1:**
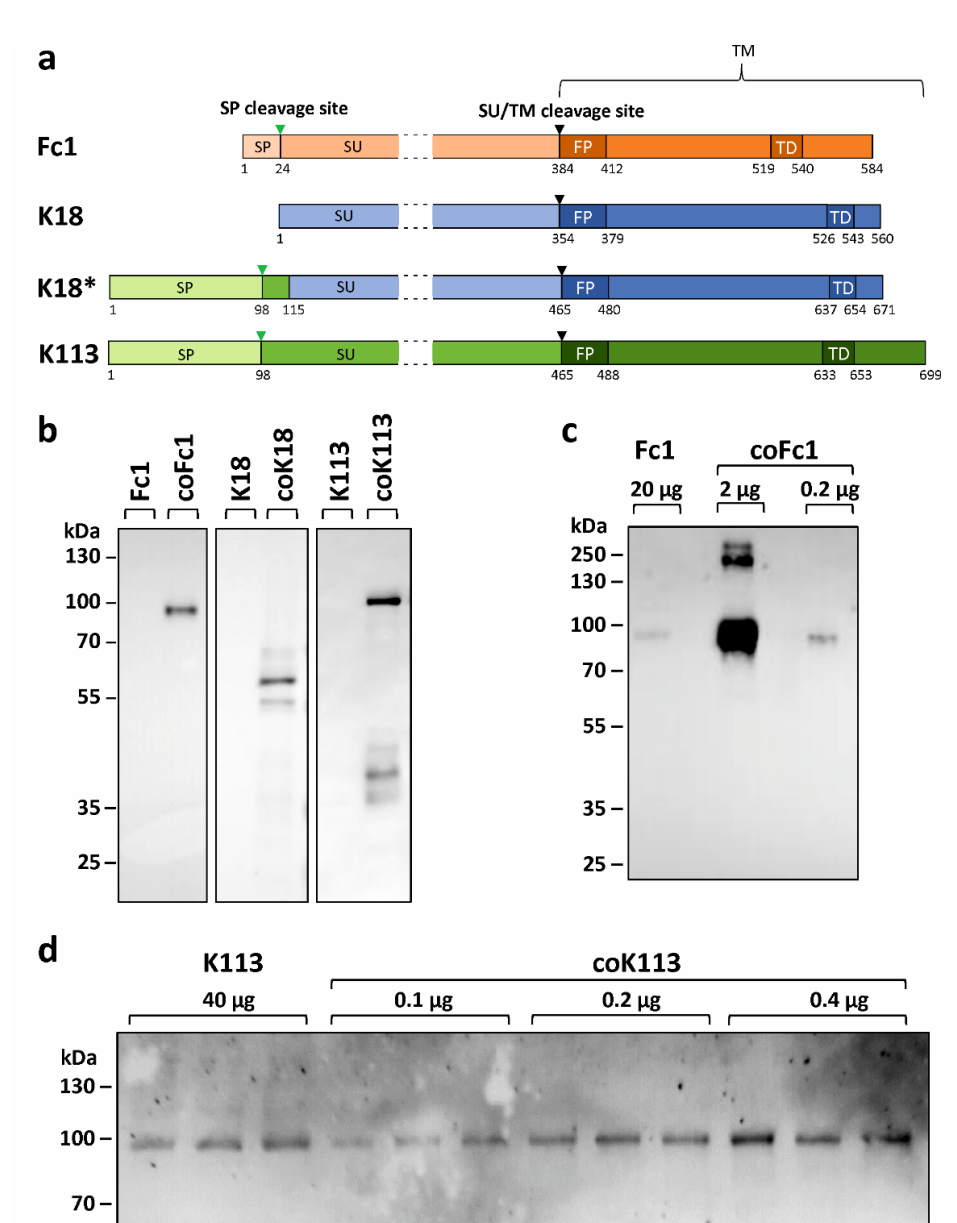
Expression and quantification of HERV envelope proteins in HEK293 cells. (**a**) Schematic representation of expressed HERV ENV. The pro-peptides are processed co- and post-translationally: The signal peptide (SP) is cleaved by signal peptidases (green triangle). The surface unit (SU) is separated from the transmembrane unit (TM) by furin-like proteases at a highly conserved consensus sequence (black triangle). The fusion peptide (FP) and the transmembrane domain (TD) are particularly hydrophobic regions within the TM. The chimeric K18* is composed of the signal peptide and another 17 amino acids of K113 plus amino acids 5–560 from K18. The numbers correspond to the amino acid positions. The SU is not shown to scale, but shortened. (**b**) Influence of codon optimization on protein biosynthesis of HERV ENV. Cell lysates were analyzed by Western blot 24 h after transient transfection of HEK293 cells with expression plasmids containing either the WT sequence or codon-optimized (co) sequences of the envelope proteins of HERV-Fc1, -K18, and -K113. Each lane was loaded with 20 µg total protein. (**c**,**d**) Representative Western blots used for densitometric quantification of expression increase caused by codon-optimization. The amount of total protein load is indicated for each lane. Representative loading controls are presented as Supplementary Figure S1. The following antibodies were used: Primary antibodies: anti-FLAG 1:1000 (Fc1), anti-HERV-K TM 1:1000 (K18, K113). Secondary antibodies: anti-rabbit IgG-HRP 1:2000, anti-mouse IgG-HRP 1:2000.

The expression of three different HERV *ENV* (Fc1, K18, K113, Figure 1a) was analyzed in transfected HEK293 cells. The expression of wild type (WT) *ENV* sequences as found in the human reference genome led to barely detectable amounts of protein for HERV-Fc1 (Figure 1b,c) and HERV-K113 (Figure 1b,d) despite application of equal protein amounts, exemplarily shown for HERV-K113 (Supplementary Figure S1). In addition, no protein could be detected for WT HERV-K18. However, when codon-optimized variants were expressed under the same conditions, all envelope proteins were readily detected (Figure 1b).

This result was corroborated by LC-MS/MS analysis of HERV-K113 and HERV-Fc1 expressing HEK293 cells. The sequence coverage, which is to a limited extent indicative for the protein concentration, coincided well with the Western blot detection of HERV proteins (Table 1, Supplementary Figure S2). The highest sequence coverage was observed for HERV proteins identified in cells with a codon-optimized protein sequence (coFc1 and coK113, Supplementary Figure S2). In cells that expressed the WT sequences, either the identified HERV protein showed only a reduced sequence coverage (K113, Supplementary Figure S2) or no HERV protein was detected at all (Fc1) by this method. In this regard, the low intracellular abundance was not due to secretion of the proteins from cells. An antibody specifically binding to the surface unit (SU) of HERV-K did not detect K18 and K113 in cell culture supernatants up to 48 h post-transfection but corroborated the intracellular accumulation of the tested proteins (Supplementary Figure S3).

For quantification of the increase in protein synthesis resulting from codon-optimization, different total protein amounts were applied to SDS-PAGE. This was necessary to enable visualization of both variants (WT vs. codon-optimized) on the same blot. Densitometric analysis of resulting proteins revealed a 150-fold (K113) or 290-fold (Fc1) increase in ENV levels caused by codon-optimization (Figure 1c,d and Supplementary Figure S4). A fold-change for K18 could not be determined, since the WT variant could not be identified by Western blot.

### 2.2. Analysis of Post-Translational Processing of HERV ENV

**Figure 2:**
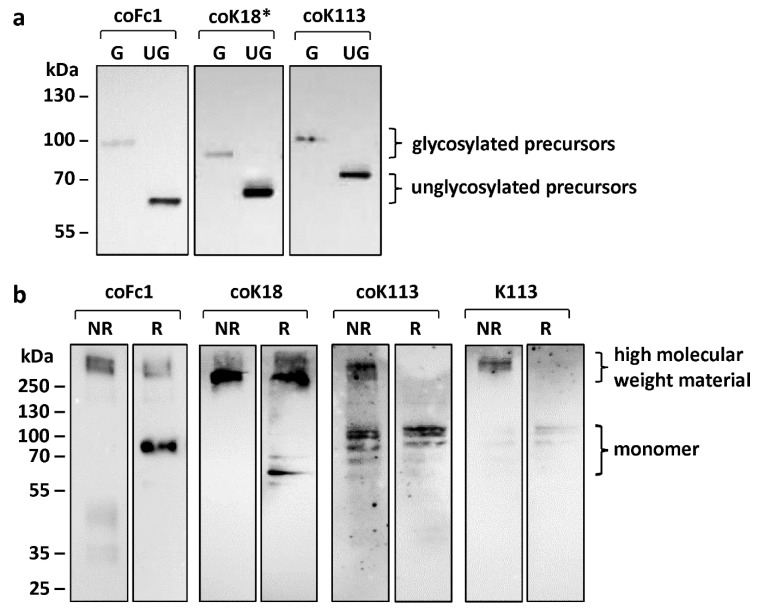
Western blot analysis for characterization of post-translational modifications of HERV ENV. (**a**) Detection of glycosylated (G) or unglycosylated (UG) envelope proteins in cell lysates of transiently transfected HEK293 cells. Notice the shift in protein size after deglycosylation by PNGase F. (**b**) Higher molecular weight material resulting from expression of HERV ENV precursors via intramolecular disulfide bridges analyzed by comparison of non-reducing (NR) and reducing (R) sample buffer conditions. The monomers are only visible when reducing (R) sample buffer was used. Either anti-FLAG antibody (coFc1) or anti-HERV-K SU HERM1821-5 antibody (coK18*, coK113) was used for detection of ENV.

Protein maturation of HERV ENV was studied in terms of N-glycosylation, cleavage of precursor proteins, and oligomerization (Figure 2). The precursors for all HERV ENV investigated in this study were detected by Western blot analysis (Figure 1b and Figure 2a).

The envelope precursors of HERV-Fc1, HERV-K18*, and HERV-K113 are glycosylated as shown by treatment of cell lysates from transfected HEK293 cells with the peptide N-glycosidase (PNGase) F (Figure 2a). The protein size after de-glycosylation corresponded to the theoretical molecular mass of ENV precursors (Fc1: 64 kDa, K113: 80 kDa, K18*: 66 kDa,). Hence, it can be concluded that these ENV are post-translationally glycosylated in the ER.

For HERV-K18, the size of the detected ENV in HEK293 cell lysates corresponded to the theoretical molecular mass of 63 kDa. In addition, treatment with PNGase F did not lead to a change in the molecular mass of the protein (Supplementary Figure S5). In contrast, HERV-K18*, which has a reconstructed signal peptide, was glycosylated. Thus, a defective signal peptide in HERV-K18 ENV prevents glycosylation due to an altered subcellular localization.

Furthermore, retroviral envelope proteins are synthesized as precursors, which are cleaved by proprotein convertases such as furin into two functional subunits (SU and TM). For all HERV ENV investigated in this study, the precursor was detected by Western blot analysis (Figure 1b and Figure 2a). For coFc1 and coK18 no cleavage of the precursor protein into SU and TM was detected. However, for coK113 we were able to visualize the appearance of the TM as detected by the TM-specific HERV-K antibody (Figure 1b, Supplementary Figure S5b). Here, additional proteins with a molecular mass of 35-40 kDa were observed. These correspond to the glycosylated TM, since after de-glycosylation proteins with the theoretical molecular mass of TM were detectable (Supplementary Figure S5b). Furthermore, protein detected by a corresponding SU-specific HERV-K antibody in lysates of HERV-K113 expressing HEK293 cells corroborated the at least partial processing of the HERV-K113 precursor into the SU and TM subunit (Supplementary Figure S5b,c). Therefore, it can be assumed that the precursor of HERV-K113 ENV is processed into TM and SU to some extent. However, the most abundant form of the envelope proteins appears to be the unprocessed precursor.

In addition, functional retroviral envelope proteins form oligomers at the plasma membrane. For all tested HERV ENV it could be shown that the expressed envelope proteins are present as an oligomer within the cell after analyzing cell lysates of transfected HEK293 cells under non-reducing (NR) conditions (Figure 2b). By omitting the reducing agent, possible disulfide bridges remain intact. Furthermore, in the non-reduced sample, the monomer did not appear, which suggests that it forms disulfide bonds with other monomers. The high-molecular double band found in these samples may therefore likely be trimers or tetramers. In contrast, under reducing conditions mainly the monomers are visible (Figure 2b), except for coFc1 and coK18. For coFc1 the oligomer band is weaker under reducing conditions but did not disappear. In addition, the oligomer band for coK18 was stable and only minor amounts of monomer were formed suggesting aggregation of the protein in the cytosol.

### 2.3. Analyzed HERV Envelope Proteins are not Transported to the Plasma Membrane but Reside within the Endoplasmic Reticulum

**Figure 3:**
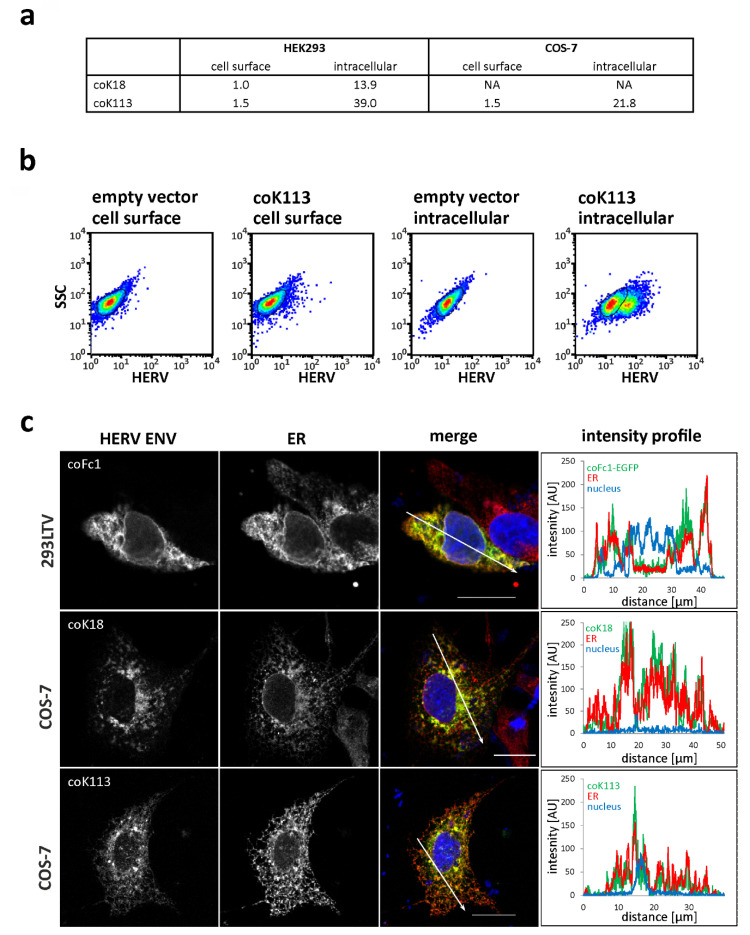
Localization of HERV ENV in transfected HEK293 cells, 293LTV cells, and COS-7 cells. (**a**,**b**) Flow cytometry of transiently transfected, non-permeabilized or permeabilized HEK293 cells and COS-7 cells stained for HERV-K ENV. The table in (**a**) shows the percentage of fluorescent cells. NA, not analyzed. In (**b**) the dot plots of HEK293 cells transiently transfected with coK113-FLAG and empty vector are shown. No staining without primary antibody was observed (Supplementary Figure S6). (**c**) Fluorescence microscopic images showing the subcellular localization of specified HERV ENV and the endoplasmic reticulum (ER) in transiently transfected COS-7 cells and 293LTV cells. The merge image is a z-projection (maximum intensity) of the three recorded channels (green: HERV ENV, red: ER, blue (DAPI): cell nucleus). Pixels with red and green fluorescence appear yellow. The graphs show a fluorescence intensity profile along the arrows drawn in the merge image. The detection of envelope proteins was performed using anti-HERV-K-TM antibody HERM1811-5 (coK18, coK113) or EGFP tag (coFc1). The ER was visualized by an anti-calnexin antibody. Scale: 20 µm.

The subcellular localization of envelope proteins from HERV-K113, HERV-K18, and HERV-Fc1 was studied using flow cytometry and immunocytochemistry. First, transfected and non-permeabilized HEK293 cells, COS-7 cells, and LN-405 cells were incubated with anti-HERV-K TM specific antibody HERM1811-5 in order to detect envelope proteins at the cellular surface. Exemplarily, transfected and non-permeabilized HEK293 cells and COS-7 cells are depicted in Figure 3a and do not reveal the presence of envelope proteins on the cellular surface (Figure 3a). Only when using permeabilized cells, a robust percentage of cells showed increased fluorescence intensities as can exemplarily be seen in the dot plot of coK113-FLAG-transfected HEK293 cells (Figure 3b). Empty vector controls stained with primary and secondary antibodies served as controls (Figure 3b). Interestingly, the percentage of ENV-producing cells depends both on the cell line (39.0% coK113-positive HEK293 cells vs. 21.8% coK113-positive COS-7 cells) and the transfected expression plasmid (13.9% coK18-positive HEK293 cells vs. 39.0% coK113-positive HEK293 cells). In this regard, shedding of the SU did not account for the low abundance on the cellular surface, since we were not able to detect K18 or K113 ENV in the cell culture supernatant using a SU-specific antibody (Supplementary Figure S3).

**Figure 4:**
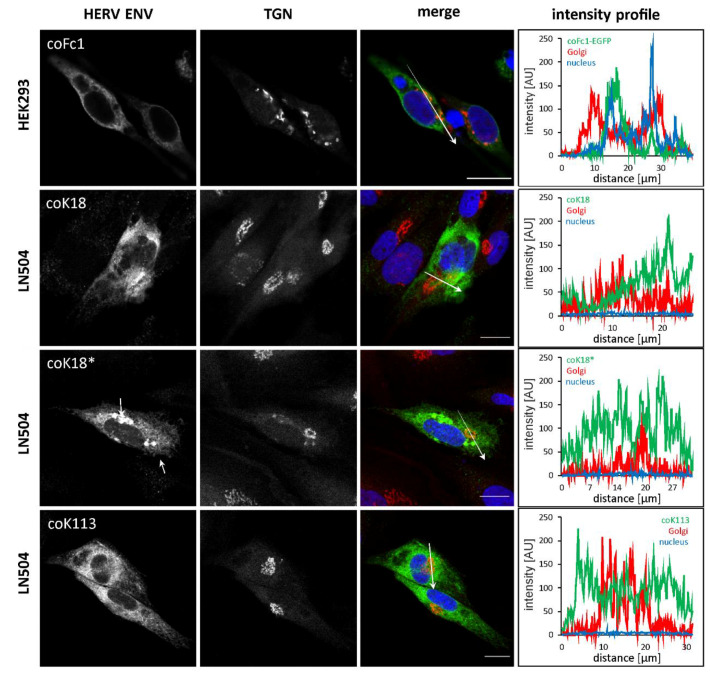
Localization of HERV ENV with trans Golgi network in transfected HEK293 and LN405 cells. Fluorescence microscopic images showing the subcellular localization of specified HERV ENV and the trans Golgi network (TGN) in transiently transfected HEK293 and LN405 cells. The merge image is a z-projection (maximum intensity) of the three recorded channels (green: HERV ENV, red: TGN, blue (DAPI): cell nucleus). Pixels with red and green fluorescence appear yellow. The graphs show a fluorescence intensity profile along the arrows drawn in the merge image. The detection of the envelope proteins was performed using anti-HERV-K-TM antibody HERM1811-5 for HERV-K and anti-FLAG for HERV-Fc1. The TGN was visualized by an anti-golgin-97 antibody for HERV-K and anti-GP73 antibody for HERV-Fc1. Scale bar: 20 µm.

To further study the localization of envelope proteins, double immunofluorescence staining of transfected and permeabilized 293LTV cells, COS-7 cells, and LN-405 cells was performed using anti-FLAG tag or anti-HERV-K TM-specific antibodies and cell organelle specific antibodies. For the envelope proteins of HERV-K18, HERV-K113, and HERV-Fc1 a similar localization in the form of branched net-like, partially vesicle- or cistern-like structures within the entire cell, excluding the cell nucleus, was observed (Figure 3c). The signal intensity was higher in areas close to the nucleus than in areas far away from the nucleus. For HERV-K18*, an unusually large number of cells with sharply defined regions of very high fluorescence intensity was observed (Figure 4 and Supplementary Figure S7). These are probably aggregated ENV within the ER lumen. The double immunofluorescence staining with the ER marker protein calnexin showed a partial overlay with the envelope protein signals, which appear yellow in the pseudo-colored Z-projection (Figure 3c). In addition to the concurrence of the signals presented in this way, intensity profiles provided evidence that the fluorescence signals of HERV ENV and ER were even colocalized, since pixels with high green (HERV ENV) fluorescence also showed high red (ER) fluorescence along a drawn distance (Figure 3c).

Furthermore, we used Golgi-specific markers GP73 for analysis of HERV-Fc1 and golgin-97 for analysis of HERV-K in order to determine the appearance of the ENV proteins in the Golgi complex. We observed only a partial overlap with Golgi markers especially for HERV-K113 (Figure 4). However, since we observed incomplete cleavage of the full-length precursor of coK113 into SU and TM (Supplementary Figure S5b,c), at least coK113 is not fully retained in the ER. Furthermore, staining using anti-tubulin corroborated the cytosolic localization of coK18 possessing a defective signal sequence (Supplementary Figure S7). In conclusion, HERV-K ENV could not be detected on the cellular surface, whereas all studied HERV ENV seem to be trapped inside the ER of transfected mammalian cells. The partial processing of the HERV-K113 precursor, however suggested only inefficient transport to the Golgi complex.

### 2.4. HERV Envelope Proteins Increase Gene Expression of Markers for Unfolded Protein Response but Trigger no Antiviral Response

**Figure 5:**
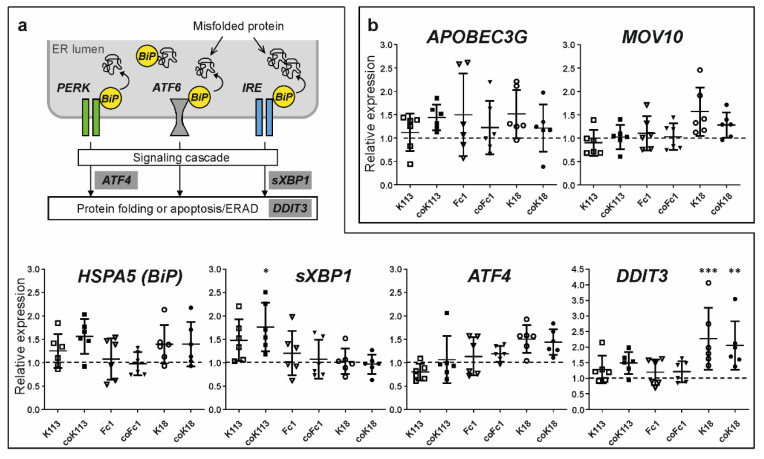
Influence of HERV ENV expression on antiviral response and unfolded protein response in transfected HEK293 cells. (**a**) The simplified scheme shows key players of the unfolded protein response (UPR). The ER chaperone immunoglobulin binding protein (BiP), which is encoded by the gene *HSPA5*, is transiently bound to three protein sensors anchored in the ER membrane: PKR-like ER kinase (PERK), activating transcription factor 6 (ATF6), and inositol-requiring enzyme (IRE). If misfolded proteins accumulate in the ER lumen, BiP selectively binds to hydrophobic regions of these proteins and detaches from the membrane sensors at the same time. Hence, they become activated via oligomerization, autophosphorylation, or glycosylation. As a result, a signaling cascade is triggered, which leads to enhanced protein folding or ER-associated degradation (ERAD). The expression normalized to *GAPDH* and relative to empty vector-transfected cells (dashed grey line) was determined. The graphs show single data points and mean values ± standard deviation. Statistics: one-way ANOVA with Dunnett’s multiple comparison test; *** *p* < 0.001; **, *p* < 0.01; * *p* < 0.05, *n* = 6. (**b**) Relative expression of genes involved in viral defense mechanisms after transfection of WT and codon-optimized HERV envelope protein sequences in HEK293 cells. Expression was normalized to *GAPDH* and empty vector-transfected cells were set as one (dashed grey line).

As HERV ENV were shown to accumulate inside the ER, it was further studied whether this induces an unfolded protein response (UPR) in transfected HEK293 cells. The UPR is part of the cellular protein quality control that facilitates correct protein folding and prevents the accumulation of misfolded proteins in the ER lumen. Using qRT-PCR analyses of transiently HERV ENV transfected HEK293 cells, the transcript levels of different genes involved in the UPR (*HSPA5, ATF4, sXBP1, DDIT3*) were determined (Figure 5a). The *HSPA5* transcript levels of coK113 were nonsignificantly elevated when compared to control vector transfected cells.

Furthermore, *sXBP1* levels were significantly increased for coK113 as well as *DDIT3* mRNA levels for K18 and coK18 compared to control vector expression (one-way ANOVA, Dunnett’s post-test, *p* < 0.05). In addition, we analyzed *HSPA5* (BiP) in WT and codon-optimized K113 and Fc1-expressing HEK cells using LC-MS/MS. As done for HERV-expression, we used sequence coverage as an indicator of protein abundance in these cells (Supplementary Figure S8). We could show that the sequence coverage and the number of proteolytic peptides increases in HERV-expressing cells compared to a vector-transfected control (Table 1). Therefore, we suggest that overexpression of HERV ENV activates UPR to some extent but focused studies for this topic are required in the future.

In addition, if a retrovirus infects a host cell, a number of cellular defense reactions occur. Among others, the group of apolipoprotein B mRNA editing enzyme, catalytic polypeptide-like 3 (APOBEC3) proteins plays an important role. These evolutionarily conserved cytidine deaminases recognize and bind retroviral single-stranded DNA to deaminate cytosine to uracil. As a result, hypermutations or degradation of viral DNA occurs. In the present study we were not able to see significant changes in *APOBEC3G* and *MOV10* expression in HEK293 cells (Figure 5b). Expression of *APOBEC3G* was always very low in HEK293 cells (Supplementary Figure S9a). Additionally, no changes in *APOBEC3B* could be detected in HERV ENV-expressing cells in comparison to empty vector-transfected cells (Supplementary Figure S9b).

Another gene activated in response to retroviral infection is *BST2*. It encodes the protein tetherin, which prevents the budding of viral particles by binding viral envelope proteins. The restriction factor *TRIM22* is also an important component of innate antiviral immunity as it recognizes viral proteins and LTRs. The transcript levels of both genes were quantified in the present study after transfection of HERV ENV expressing plasmids in HEK293 cells. No differences in comparison to the control were found (Supplementary Figure S9b).

### 2.5. Rare Leucine and Valine Codons Result in Decreased ENV Protein Synthesis in Human Cell Lines

**Figure 6:**
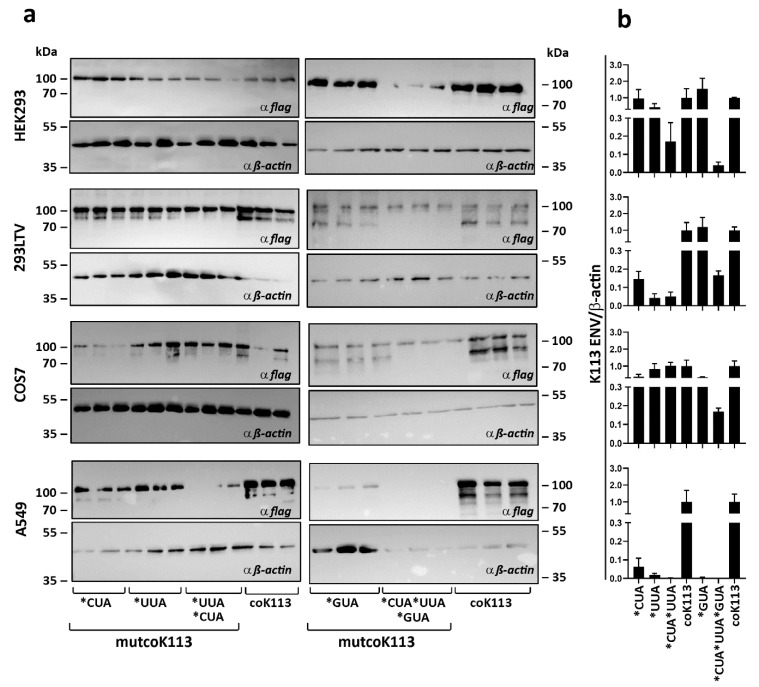
Expression of coK113 ENV mutants in mammalian cells. (**a**) Three human cell lines (HEK293, 293LTV, and A549) and monkey COS7 cells were transfected with expression vectors containing codon-optimized (coK113) or rare codon mutants of this sequence (mutcoK113). After 24 h the cells were harvested. Total amount of 20 µg protein from three independent experiments was analyzed by Western blotting. For detection of ENV, staining against their C-terminal FLAG tag was performed. As reference the expression of β-actin was observed. (**b**) Densitometric analysis of normalized expression level is shown. The expression of ENV precursors was measured and normalized to expression of β-actin by densitometric analysis using ImageJ software. The complete codon-optimized ENV was set as 1. Mean and standard errors were calculated and visualized by GraphPad Prism5.

The limited expression of native ENV raised the question of a possible mechanism that might be involved in increased protein synthesis after codon-optimization. Interestingly, the wild-type envelopes of HERV-K family members contained a high number of rare leucine (CUA and UUA) or valine (GUA) coding triplets. Those codons can be seen as rare, because they are less served through the human tRNA pool. In the sequence of codon-optimized K113 ENV 43 of these codons were changed to the more common CUG or GUG triplets, respectively. The generation of different codon-optimized variants of K113 ENV with partial back-mutation to respective native codons for leucine or valine (mutcoK113) should lead to a decrease in protein synthesis through the limited availability of tRNAs. The expression of the complete codon-optimized K113 ENV (coK113) and five mutants including different rare codons (7× CUA; 23× UUA; 30× CUA and UUA; 13× GUA; 43× CUA and UUA and GUA) was analyzed in four mammalian cell lines, three from human origin, HEK293, 293LTV and A549, and one originating from African green monkey, COS-7. The results of Western blots of three independent transfection experiments of each vector normalized to expression of β-actin by densitometric analysis are shown (Figure 6).

Assuming coK113 ENV levels as 100%, alterations for leucine and valine codon mutants were calculated. In all human cell lines, we observed decreasing K113 ENV level with increasing number of rare leucine (in total 30) codons with strongest drop in A549 cells (1/500th for mutcoK113*CUA*UUA). Interestingly, the usage of rare leucine codons alone showed no influence on expression of ENV in COS-7 cells. Here, we first observed decreasing protein level after introduction of rare valine codons (mutcoK113*GUA: 3-fold; mutcoK113*GUA*CUA*UUA: 5.6-fold). The vector with back mutations of rare valine codon GUA led to decrease of protein synthesis in A549 cells (1/200th), whereas in contrast no effect was measured in HEK293 cells or 293LTV cells. The vector containing all 43 rare codons (mutcoK113*GTA*CTA*TTA) showed lower expression of K113 ENV in all three human cell lines (HEK293: 13.5-fold; 293LTV: 1/6th), with no detectable protein in A549 cells.

### 2.6. Rare Codons have a Negative Effect on Expression of HERV-K113 Envelope Protein in a Cell-Free Expression System

**Figure 7:**
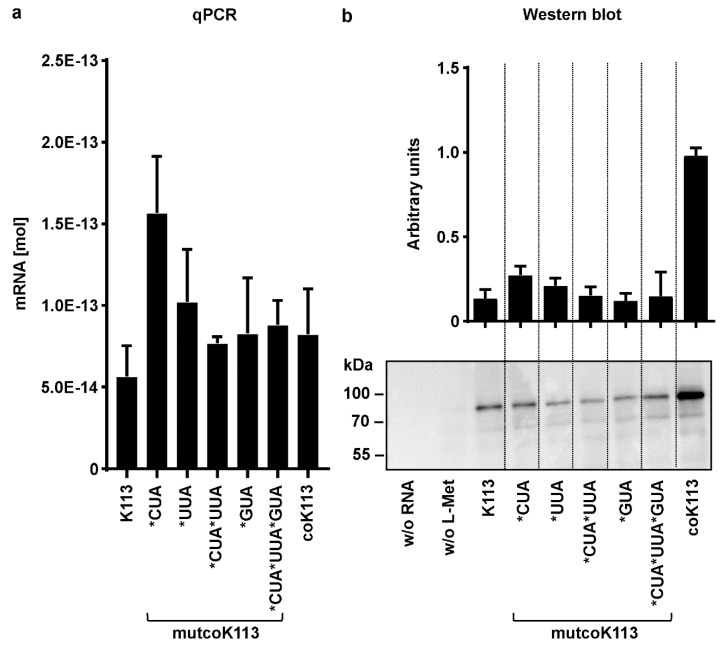
Cell-free expression of HERV-K113 envelope variants in qRT-PCR and Western blotting analysis. K113 envelope sequences of native (K113), codon-optimized (coK113), or its mutants with rare codons (mutcoK113) were in-vitro transcribed into mRNA and then translated into protein in a cell-free expression system. Mean and standard errors were calculated from at least two (four of native and codon-optimized K113 envelope) individual experiments with GraphPad Prism5. (**a**) In total, 1 µg of linearized expression vector DNA was used for generation of mRNA by transcription starting at T7 promotor site upstream of ENV by RNA polymerase. A total of, 1 µg of mRNA was reverse transcribed into cDNA and used as template in qRT-PCR. The calculation of absolute mRNA levels was performed using standard curves with native or codon-optimized vector DNA, respectively. (**b**) In total, 4 μg of transcribed mRNA of ENV in (**a**) were translated into protein using reticulocyte lysate from New Zealand white rabbits including a mixture of all components necessary for translation. A translation reaction without RNA and a sample of codon-optimized RNA without amino acid methionine (L-Met) served as negative controls. An equal aliquot of all reactions was loaded for Western blotting. Densitometric analysis was performed by using ImageJ software, n=4. The de-glycosylated precursor of K113 ENV was observed at 80 kDa using anti-HERV-K-TM (HERM1811-5) antibody.

In addition to expression in mammalian cells, cell-free synthesis of K113 *ENV* sequences was performed to minimize the influence of environmental factors on efficiency and reproducibility, such as e.g., cell stress or days in culture. The quantification of absolute mRNA levels using standard curves of WT or codon-optimized DNA vectors in qRT-PCR resulted in lower amounts of transcripts for the WT *ENV*, with 5.8 × 10^−14^ mol, compared to all codon-optimized variants, on average approx. 8 × 10^−14^ mol (Figure 7a).

The transcript levels of mutcoK113 variant with back-mutated leucine codons CUA were increased (1.5 × 10^−13^ mol). In cell-free protein synthesis with rabbit reticulocyte lysate, the full-length and de-glycosylated ENV precursor could be detected for all K113 ENV variants at 80 kDa using an equal aliquot of all reactions in Western blotting. The expression of coK113 was increased by six times compared to the native ENV shown by densitometric analysis of four individual experiments. Mutants of coK113 ENV expressed protein levels comparable to those of native ENV in two individual experiments (Figure 7b and Supplementary Figure S10).

Although the transcript levels of the variants are almost equal to or higher than those of coK113, protein biosynthesis seems to be impaired in all of these variants.

### 2.7. Inefficient Synthesis of Native HERV ENV—A Problem of Secondary RNA Structure?

The weak synthesis of native HERV ENV prompted us to investigate the folding of mRNA secondary structures as a possible reason.

**Figure 8:**
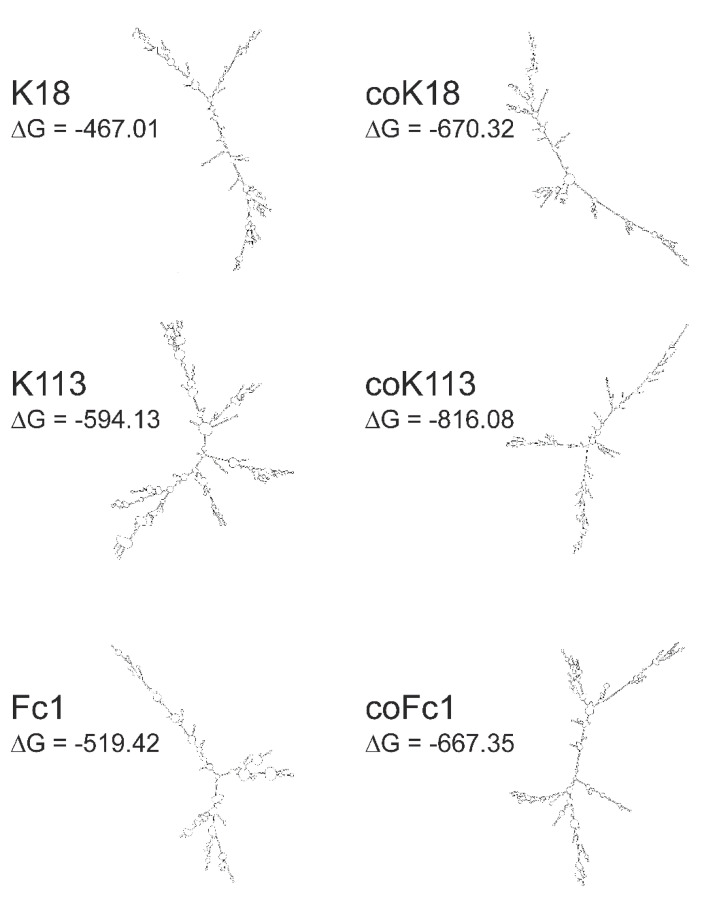
In-silico models of mRNA secondary structure of cloned native and codon-optimized HERV envelope RNAs. For all RNAs the sequence between the predicted transcription start site and the poly(A) site from the vector pcDNA3.1(+) was used for modeling. Models were calculated using *The mfold Web Server* (http://unafold.rna.albany.edu/?q=mfold, last accessed data 14.02.2020). For all RNAs the calculated structure with the lowest ΔG is shown.

The secondary structures of WT and codon-optimized HERV ENV mRNAs of Fc1, K18 and K113 were predicted using *The mfold Web Server* (http://unafold.rna.albany.edu/?q=mfold; last accessed data 14.02.2020). The in-silico models of envelope RNAs revealed differences in the secondary structure of native mRNAs compared to their codon-optimized counterparts (Figure 8). Interestingly, the calculated minimum free energy (ΔG) of all codon-optimized sequences was lower than that of WT sequences. The difference of ΔG of WT and codon-optimized versions was 218.95 for K113, 203.31 for K18, and 147.93 for Fc1, respectively.

## 3. Discussion

In the present study, we investigated the expression of three selected ENV, which are frequently associated with inflammatory diseases and cancer.

First, we observed limited expression of native ENV by transient transfection in mammalian cells (see Figure 1b–d). Codon optimization of sequences increasing ENV expression up to 150-fold (K113) and 290-fold (Fc1) was essential for further analysis of maturation, glycosylation and localization. In 2009, Hanke and colleagues also observed a 50-fold increase by codon optimization for K113 ENV [36]. This deviation is probably a result of different optimization algorithms and due to use of an artificial envelope sequence, which according to the authors corresponds to the suggested K113 *ENV* at the time of integration [36]. Interestingly, the reconstructed ENV showed increased proteolytic cleavage in SU and TM and additional glycosylation sites at the TM compared to the K113 ENV in our study [36]. We found that the majority of detected K113, Fc1, K18 and K18* ENV in all cell lysates is composed of the unprocessed ENV precursor (Figure 2a and Supplementary Figure S1). With exception of K18 all studied ENV were glycosylated and the protein sizes after de-glycosylation corresponded to the theoretical molecular mass of ENV precursors (K113: 80 kDa, K18*: 66 kDa, Fc1: 64 kDa). Of particular interest is that glycosylation of K18 ENV precursor could be rescued by reconstruction of a signal peptide sequence in K18* ENV (Figure 2a). Therefore, it can be suggested that in type 1 proviruses of the HERV-K (HML-2) family, e.g., K18, the loss of the signal peptide sequence by the characteristically 292 bp deletion in the *POL*-*ENV* region, prevents glycosylation by an altered subcellular localization.

In double immunostaining experiments by antibody labelling of organelle marker proteins, we demonstrated localization of HERV-K18, -Fc1, and -K113 ENV inside the ER (Figure 3c). The appearance especially of HERV-K113 ENV in the compartment of the Golgi can also be suggested due to (I) a partial overlap of fluorescence signals of the applied trans-Golgi network marker and ENV (Figure 4) and (II) the cleavage of precursor proteins into subunits SU and TM as shown for HERV-K113 (Figure 1b, Supplementary Figure S5b,c). The cleavage by furin proteases, which are active in the Golgi apparatus, is essential in protein maturation of retroviruses promoting membrane fusion and infectivity [37,38]. Our results for K113 ENV are in line with previous reports, detecting a reduced surface expression in comparison to K108 ENV [39]. Our study suggests the absence of K113 ENV at the plasma membrane as detected by immunohistochemistry and flow cytometry (Figure 3) and could explain absence of K113 ENV in pseudotyping experiments using simian immunodeficiency virus [39]. In this regard, an inaccessibility of the utilized antibody to its epitope is unlikely, since prior reports using the same antibody showed HERV-K ENV on the cellular surface of transfected HeLa cells and human PBMCs [39,40]. The accumulation of HERV ENV inside the ER was further supported by investigating the induction of genes involved in the unfolded protein response (UPR). Overexpression of proteins after codon-optimization lead to a strong enrichment of protein in the ER promoting misfolding [41]. As a result, coK113 ENV and K18/coK18 ENV showed elevation of e.g., *sXBP1* and *DDIT3* (Figure 5). In addition, LC-MS/MS experiments suggested a higher abundance of *HSPA5* (BiP) after expression of K113/coK113 and Fc1/coFc1 (Table 1). The physiological relevance of the observations remains elusive, since it is poorly understood to what extent the expression of dormant proviral sequences can be reactivated in the human genome. The applied codon-optimization would mimic a stronger activation according to the low level of protein synthesis from native sequences. Therefore, we suggest that the lack of efficient intracellular trafficking is (I) due to the defective signal sequence in K18 or (II) inefficient cleavage of the precursor as shown for coK113. In this regard, it has been shown previously, that inefficient cleavage of the ENV precursor interferes with intracellular trafficking, e.g., as shown for bovine HERV-K [42]

In accordance to that, we also investigated the altered nucleotide sequence during codon-optimization of ENV. The majority of optimized nucleotides in the ENV of K113 belonged to triplets coding for leucine (CUA and UUA) or valine (GUA) indicating that the low protein amounts of native ENV might be due to a limited availability of appropriate cognate t-RNAs. Interestingly, we observed that gradually elevation of rare leucine codons in the codon-optimized *ENV* led to a decreased ENV expression in human cell lines (Figure 6). With the exception of A549 cells, the re-introduction of rare valine codons only caused a reduced protein expression in the monkey cell line, COS7. Thus, a different tRNA pool in the applied human and monkey cells might be part of an explanation. Furthermore, we decided to investigate expression of all K113-*ENV* variants in a cell-free system, which offers the advantage of a controlled transcription and translation process. As a result, we detected the expression of all K113 ENVs as de-glycosylated precursor with a molecular mass of 80 kDa (Figure 7b and Supplementary Figure S10). Interestingly, the native ENV could be detected next to the codon-optimized ENV by loading equal amounts of protein lysate, which implicates that the protein yield of native ENV was much higher in the cell-free system than in the mammalian cells. In this regard it is of interest that we observed a discrepancy in transcript amounts between the native and all codon optimized *ENV* sequences by using equal RNA amounts for synthesis of cDNA and equal amounts of cDNA performing qRT-PCR, respectively (Figure 7a). In-silico prediction of mRNA secondary structures using the *mfold Web Server* algorithm revealed (I) an apparently different modelling of native and codon-optimized *ENV* and (II) a lower minimum free energy of all codon-optimized sequences indicating a higher thermodynamic stability of these structures (Figure 8). These increased thermodynamic stabilities might influence translation efficiency. In addition, Zhang and colleagues observed that stable RNA secondary structures, e.g., stem loops, could be skipped by the reverse transcriptase, resulting in shortened transcripts and early termination of cDNA synthesis [43]. The algorithm used for codon optimization (see Section 4) considers not only the codon usage bias but also multiple parameters influencing transcriptional and translational efficiency, e.g., the GC content or the presence of RNA destabilizing elements. Interestingly, codon composition has been shown to directly influence mRNA stability. The impact of codon usage on stability varies between species or cell types [44] and cannot be predicted certainly today. Moreover, the tRNA pool seems to be dynamic [45] and not all cells of a given species must behave identically. Codon usage has also been shown to influence transcription independent of RNA stability [46,47]. Finally, mRNAs are subjected to epitranscriptomic modifications that influence RNA stability and gene expression, which has been studied especially in viruses [48,49]. All these factors might be responsible for the different impact of codon composition on HERV ENV expression in different cell lines. The complexity of secondary structures of native *ENV* should be investigated in future analysis.

## 4. Materials and Methods

### 4.1. Generation of Expression Plasmids

The cDNA for HERV-Fc1 *ENV* was isolated from the human embryonic testicular carcinoma cell line H12.5 [50]. The obtained sequence contained a silent mutation C705T compared to the sequence of the reference sequence (NCBI accession no.: 105373297), which was mutated back via site-directed mutagenesis. For subsequent cloning in expression vectors, a restriction site for *Nco*I was mutated so that the silent mutation A438T was inserted (used primers are specified in Supplementary Table S1). The sequences of the envelope proteins of HERV-K18.2 (NCBI accession no.: 100775105) and HERV-K113 (NCBI accession no.: 17099689) were synthesized de novo (Eurofins Genomics, Ebersberg, Germany; GenScript, Piscataway, NJ, USA). Codon-optimized gene sequences for all three envelope genes were generated using the OptimumGene algorithm (GenScript, Piscataway, NJ, USA). Additionally, K18* comprising a functional signal peptide was generated by combining the codon-optimized signal sequence of HERV-K113 with codon-optimized HERV-K18. All sequences contained a 3′-FLAG tag sequence. In addition, for HERV-Fc1, also an EGFP was fused C-terminally. Amplification of the target sequences was accomplished by conventional PCR using Phusion High Fidelity Polymerase (New England Biolabs, Ipswich, MA, USA) according to the manufacturer’s protocol. All used primer sequences are listed in Supplementary Table S1. The PCR product was purified using the Wizard^®^ SV Gel and PCR Clean-Up System (Promega GmbH, Mannheim, Germany). After restriction digestion of the purified PCR product and the expression vector pcDNA3.1(+) (Thermo Fisher Scientific, Waltham, MA, USA) using appropriate restriction enzymes (New England Biolabs, Ipswich, MA, USA) as specified in Supplementary Table S1, ligation was performed using T4 DNA Ligase (Thermo Fisher Scientific) according to the manufacturer’s protocol and transformed into *E. coli* DH5alpha. Thereafter, the DNA of one overnight colony grown in 1 mL LB medium supplemented with selection antibiotics was isolated using GeneJET Plasmid Miniprep Kit (Thermo Fisher Scientific) according to the manufacturer’s protocol. The corresponding expression vectors are designated as follows: Fc1-FLAG; Fc1-EGFP; coFc1-FLAG; K18-FLAG; coK18-FLAG, coK18*-FLAG, K113-FLAG; coK113-FLAG, where “co” stands for codon-optimized.

### 4.2. Cell Lines

The human embryonic kidney cell line HEK293 [51] and the African green monkey cell line COS-7 [52] was obtained from the *Deutsche Sammlung für Mikroorganismen und Zellkulturen (DSMZ)*, Braunschweig, Germany). 293LTV cells were obtained from Cell Biolabs, San Diego, CA, USA. The human glioblastoma cell line LN-405 [53] was a kind gift from I. Schulz, Probiodrug AG, Halle, Germany. The human lung adenocarcinoma cell line A549 [54] was obtained from CLS, Eppelheim, Germany. HEK293 cells, LN-405 cells, and COS-7 cells were grown in DMEM (Life Technologies, Carlsbad, CA, USA), supplemented with 10% FBS (Life Technologies, Carlsbad, CA, USA). 293LTV cells were grown in DMEM, supplemented with 10% FBS, 1x GlutaMAX (Life Technologies, Carlsbad, CA, USA) and 1x non-essential amino acids (Life Technologies, Carlsbad, CA, USA). A549 cells were cultivated in RPMI1640 medium supplemented with 10% FBS. All cell lines were cultivated at 37 °C with 5% CO_2_ (HEK293, 293LTV, COS-7) or 10% CO_2_ (LN-405).

### 4.3. Transfection

Unless otherwise stated, cells were seeded at 1.4 × 10^6^ cells/well (HEK293 cells), 1.2 × 10^6^ cells/well (293LTV cells), 0.4 × 10^6^ cells/well (COS-7 cells), or 1.3 × 10^6^ cells/well (A549 cells) in 6-well plates. After 24 h, transfection with the appropriate vectors using Opti-MEM reduced-serum medium (Life Technologies, Carlsbad, CA, USA) and Lipofectamine 2000 (Life Technologies, Carlsbad, CA, USA) was carried out according to the manufacturer’s protocol. In total, 4 µg vector and 7.5 µL Lipofectamine 2000 were used for each well. Empty vector-transfected cells served as controls. The transfected cells were incubated for 24 h before further use.

### 4.4. Protein Extraction

Cells were detached from the culture flask by incubation with trypsin (Life Technologies, Carlsbad, CA, USA) and separated from the cell culture medium by centrifugation (300× *g*, 5 min). The cell pellet was washed twice with 0.5 mL cold PBS (300× *g*, 5 min). The cells were then resuspended in 200 µL cell extraction buffer (CEB, Life Technologies, Carlsbad, CA, USA) supplemented with 10 mM AEBSF Protease Inhibitor (Sigma Aldrich, St. Louis, MO, USA) and 1x Complete Mini EDTA-free Protease Inhibitor Cocktail (Roche Diagnostics GmbH, Basel, Switzerland) and incubated on ice for 30 min with regular mixing. After centrifugation at 13,000× *g* for 30 min at 4 °C, the cell lysates were transferred to new reaction tubes. The concentration was determined by UV/VIS spectrophotometry using Bradford reagent. For LC-MS/MS analysis, pellets of HERV-expressing HEK293 cells were dissolved in 75 µL of 0.1% (*w/v*) SDS (in 50 mM NH_4_HCO_3_, pH 8.0), incubated for 10 min at 95 °C, and chilled on ice for 5 min. Then, 75 µL of cell extraction buffer (Invitrogen, FNN0011) were added and samples were again incubated on ice for 30 min and subsequently treated with ultrasound for 2 min. Cell debris were removed by centrifugation (10 min with 16,000× *g* at 4 °C) and 100 µL of the supernatant were mixed with 25 µL of 5 × SDS sample buffer.

### 4.5. Deglycosylation

Deglycosylation was carried out using the PNGase F Deglycosylation Kit (New England Biolabs, Ipswich, MA, USA) on 20 µg total protein lysates under denaturing reaction conditions according to the manufacturer’s protocol. A reaction mixture without PNGase F served as control.

### 4.6. SDS-PAGE

SDS polyacrylamide gel electrophoresis (PAGE) was performed according to Laemmli [55]. The polyacrylamide gels used contained a stacking gel fraction (5% acrylamide) and a separating gel fraction (10% or 12% acrylamide). The samples were mixed with reducing sample buffer and boiled at 95 °C for 10 min before application. Electrophoretic separation was performed at 90 V (20 min), followed by 140 V.

For LC-MS/MS analysis, samples were incubated for 5 min at 95 °C and subsequently allowed to cool down at room temperature. Then, 50 µL of each sample were applied on a 6% stacking gel and run with 60 V for 25 min. Proteins were stained with Coomassie Brilliant Blue for 30 min at room temperature and de-stained with MeOH/H_2_O/acetic acid (500/400/100; *v/v/v*) for 2 h (Supplementary Figure S11).

### 4.7. Western Blot Analysis

SDS gels, the nitrocellulose membrane (GE Life Sciences, Freiburg, Germany), and four filter papers were soaked in Towbin Buffer (0.025 mol/L TRIS pH 8.3; 0.192 mol/L glycine; 20% (*v*/*v*) methanol) for at least 20 min. Protein transfer to the nitrocellulose membrane was performed with a semidry blot system under a constant electrical voltage of 15 V for 45 min. The membrane was then incubated for 1 h in blocking buffer (5% (*w*/*v*) milk powder in TBS-T), followed by incubation with the appropriate primary antibody in blocking buffer at 4 °C for 12-16 h. For detection of the FLAG tag, anti-DYKDDDDK antibody (Cell signaling, Cambridge, Great Britain) diluted 1:1000 was used. For detection of the HERV-K, TM subunit anti-HERV-K TM HERM1811-5 and anti-HERV-K SU HERM1821-5 (Austral Biologicals, San Ramon, CA, USA) diluted 1:1000 were used. Please refer to Supplementary Table S2 for a complete list of applied antibodies. Thereafter, the membrane was washed three times in TBS-T for 10 min each before incubation with secondary antibodies anti-rabbit IgG-HRP (Cell signaling, Cambridge, Great Britain) or anti-mouse IgG-HRP (Cell signaling, Cambridge, Great Britain) diluted 1:2000 for 1 h at room temperature. After two washes in TBS-T and one wash in TBS, immunodetection by chemiluminescence was performed using the SuperSignal West Pico/Femto Chemiluminescent Substrate Kit (Thermo Fisher Scientific) according to the manufacturer’s instructions. The signal strength of the chemiluminescent signals was recorded using the Fusion FX-7 imager (Vilber Lourmat Deutschland GmbH, Eberhardzell, Germany).

### 4.8. Sample Preparation for Mass Spectrometry

Proteins from SDS gels were excised and Coomassie Brilliant Blue was removed by repeated incubation (five cycles at 30 min) in 100 mM NH_4_HCO_3_ (in H_2_O, pH 8.0) and 100 mM NH_4_HCO_3_ (in ACN/H_2_O (500/500; *v/v*), pH 8.0) at 37 °C. Gel bands were dehydrated by incubation in neat ACN for 10 min and finally dried by rotational vacuum concentration (10 min at ≈30 °C). Disulfide bonds were reduced by incubation in DTT solution for 45 min at 50 °C. Samples were allowed to cool down and DTT was exchange for iodoacetamide solution. Alkylation of cysteine residues was carried out for 1 h at room temperature in the dark. The iodoacetamide solution was discarded and gel bands were washed 2x with 100 mM NH_4_HCO_3_ followed by dehydration in neat ACN for 10 min and drying by rotational vacuum concentration (10 min at ≈30 °C).

Trypsin (Promega, V5280) stock solution (c = 1 µg/µL) was thawed on ice and diluted 1:50 with 40 mM NH_4_HCO_3_ (in H_2_O/ACN (900/100; *v/v*), pH 8.0). Gel bands were incubated for 1 h at room temperature with 75 µL of digestion solution. The latter was removed and 150 µL of 40 mM NH_4_HCO_3_ (in H_2_O/ACN (900/100; *v/v*), pH 8.0) were added. Gel bands were incubated for 8 h at 37 °C. Afterwards, proteolytic peptides were extracted by incubation of the gel bands in H_2_O with frequent vortexing and treatment with ultrasound for 1 min. This extraction step was repeated with ACN/H_2_O/TFA (500/450/50; *v/v/v*) and neat ACN. Digestion buffer and extraction solutions of each sample were combined in one tube and subsequently concentrated by rotational vacuum concentration (1 h at ≈30 °C). Concentrated gel extracts were purified with C_18_ stage-tips (10 layers). Elution was carried out with 10 µL of ACN/H_2_O/FA (600/390/10; *v/v/v*).

### 4.9. LC-MS/MS Data Acquisition and Analysis

Samples were analyzed on an Ultimate 3000 RSLC nano-HPLC system coupled with an Orbitrap Fusion mass spectrometer (Thermo Fisher Scientific). Chromatography was performed by applying 240-min gradients with reversed phase C18 columns (µPAC 900 nL C18 trapping column and µPAC™ 50 cm C18 chip-based separation column, Pharmafluidics). For MS data acquisition, a data-dependent top 5s method was used. FTMS survey scans were acquired in the m/z range 300–1700 (R = 120,000 at m/z 200). MS/MS scans of the most abundant signals of the survey scan were acquired in parallel by FTMS and ITMS. For FTMS higher energy collision-induced dissociation (HCD) was used with 27% normalized collision energy (NCE), an isolation window of 1.5 Th and an intensity threshold of 30,000. For ITMS collision induced dissociation (CID) was used with a NCE of 35%, an isolation window of 2.2 Th, and an intensity threshold of 5000). Dynamic exclusion was enabled and exclusion time was set to 60 s.

Data analysis was carried out with PEAKS Studio (version 7.5) using the human sequences of the UniProt/SwissProt database (release 2019_11). Refinement of raw mass spectrometry data included merging of MS/MS scans (0.1 min retention time window, 5.0 ppm precursor *m/z* error tolerance, merging of CID and HCD scans was enabled) and precursor mass correction. The database search was performed using the following mass tolerances: 5.0 ppm for MS and 20 mDa for MS/MS. The maximum number of both, post-translational modifications per peptide and missed cleavage sites, was 3. Non-specific proteolytic cleavage was allowed for both termini. The FDR value was set to 0.01%.

### 4.10. Flow Cytometry

A total of 24 h after transfection the cells were washed with PBS and incubated with Accutase (PAA Laboratories, Pasching, Germany) for 5 min at room temperature to remove them from the cell culture vessel. Flow cytometry was performed essentially using standard methods. In short, cells were incubated with anti-HERV-K TM HERM1811-5 (diluted 1:500 in staining buffer) for 45 min on ice. Goat anti-mouse Cy2 (Supplementary Table S3), diluted 1:200 in staining buffer, was used as secondary antibody for 45 min on ice in the dark. After two washes with staining buffer the cell pellet was finally resuspended in 100 µL PBS. For intracellular staining, the protocol was essentially the same except for fixation (4% PFA) and permeabilization (0.1% saponin in PBS) steps before application of the primary antibody. The samples were analyzed using a FACSCalibur (Becton Dickinson, Franklin Lakes, NJ, USA). For each sample 10,000 cells were counted. Data analysis was performed with the FlowJo v10.3 software (Becton Dickinson, Franklin Lakes, NJ, USA).

### 4.11. Immunocytochemistry

Cells were seeded at 7 × 10^4^ cells/well (293LTV cells) or 4 × 10^4^ cells/well (LN-405 cells, COS-7 cells) in chamber slides and 24 h later cells were transfected with appropriate vectors as described before using 0.7 µg vector and 2 µL Lipofectamine 2000 per well. The following day the cells were washed with PBS (Life Technologies, Carlsbad, CA, USA) and fixed in 4% PFA in PBS for 15 min at room temperature. This was followed by a PBS washing step and a 5-min incubation with 50 mM ammonium chloride in PBS to quench aldehyde autofluorescence. The cells were then permeabilized for 10 min with 0.1% saponin in PBS (PBS-S), incubated for 30 min in blocking buffer (0.1% saponin, 3% goat serum (Life Technologies, Carlsbad, CA, USA) in PBS), and incubated overnight at 4 °C with the appropriate primary antibody diluted in blocking buffer. The cells were then washed twice with PBS-S and incubated for 1 h with the appropriate secondary antibody in blocking buffer. After two washes with PBS-S and PBS each, the cells were incubated for 2 min in the dark with DAPI staining solution (5 ng/mL, Molecular Probes, Eugene, OR, USA) and then washed twice with PBS. Finally, the cells were embedded in Citifluor AF1 mounting medium (Citifluor, Hatfield, PA, USA) and sealed with cover glasses. Unless otherwise indicated, all incubation steps were performed at room temperature. If the localization of HERV ENV on the cell surface was to be checked, the permeabilization step and the use of saponin were omitted in all subsequent steps. If a HERV ENV coupled to EGFP was transfected, the cells were only fixed as described above, incubated with ammonium chloride, and DAPI stained before they were cover slipped. All used antibodies with corresponding dilutions are listed in Supplementary Table S3. The fluorescence images were taken with a 40× or 63× water immersion objective using a laser scanning microscope LSM 780 (Carl Zeiss AG, Oberkochen, Germany).

### 4.12. RNA Extraction and cDNA Generation

Total RNA was isolated using the NucleoSpin XS kit (Macherey-Nagel, Düren, Germany) as specified by the manufacturer. Transcription into complementary DNA (cDNA) was accomplished by mixing 200 ng total RNA with 1 µL of 100 µM random hexanucleotide primers (Roche Diagnostics GmbH, Basel, Switzerland), 1 µL of 10 mM dNTPs (Thermo Fisher Scientific), filled to 14 µL with nuclease-free water and incubated for 5 min at 65 °C. Then, 4 µL first-strand 5 × buffer (Thermo Fisher Scientific), 2 µL of 100 mM DTT solution (Thermo Fisher Scientific), 0.5 µL SuperScript II reverse transcriptase (Life Technologies, Carlsbad, CA, USA), and 1.5 µL nuclease-free water were added. The preparation was incubated for 10 min at 25 °C, followed by 50 min at 42 °C and finally 15 min at 70 °C. For quantification of in vitro transcribed HERV *ENV*, 1 µg RNA was diluted in 16 µL nuclease-free water and 4 µL of cDNA mix (qScript cDNA Synthesis Kit, QuantaBio), containing reverse transcriptase, enzymes, and buffer, was added. Synthesis was done in three steps: 5 min at 22 °C, 30 min at 42 °C, and 5 min at 85 °C. The transcribed cDNA was stored at −20 °C.

### 4.13. Quantitative Real Time PCR (qRT-PCR)

Quantification of gene expression by qRT-PCR was performed using PowerUP SYBR Green Mastermix (ThermoFisher, Germany). Each reaction contained 12.5 ng of cDNA, 500 nM of forward and reverse primer, 5 µL 2 × PowerUP SYBR Green Mastermix and 4 µL of water. Used primer sequences are listed in Supplementary Table S4. For amplification a QuantStudio3 cycler (ThermoFisher) with QuantStudio Design and Analysis Software v.1.4.3 was used. After an initial denaturation step at 95 °C for 10 min, amplification was performed by 40 cycles with denaturation at 95 °C for 15 s and primer annealing and extension at 60 °C for 60 s. Two to three technical replicates were measured for each sample in six independent experiments (biological replicates). *GAPDH* was used as reference gene for normalization and quantification was performed according to the 2^(-Δ(Δ)Ct)^ method [56]. To quantify in vitro transcribed RNA qRT-PCR, GoTaq qPCR Master Mix (Promega, Walldorf, Germany) was used. In this case, a 20 µL-reaction with 10 µL (2×) SybrGreen mix, 1250 nM forward and reverse primer, nuclease-free water, and 1 µL cDNA was mixed. Standard curves with serial dilutions of plasmid DNA containing WT or codon-optimized K113 *ENV* sequences were used for calculation of absolute amounts. As reference, the amplification of a vector specific target (neomycinR) was analyzed.

### 4.14. Cell-Free In Vitro Transcription and Translation

All expression vectors containing K113 *ENV* sequences were digested after the poly(A) site using restriction enzyme *Sma*I (10 U/µL, ThermoScientific) at 30 °C for 4 h. After inactivation at 65 °C for 20 min, the reaction was loaded with 6x DNA loading dye on a 1% agarose gel and the linearized vector was eluted with GeneJet Gel Extraction Kit (ThermoScientific) according to manufacturer’s protocol. In vitro transcription starting at the T7 promotor was performed with 1 µg linearized vector according to the manufacturer’s protocol (MEGAscript T7 Kit, ThermoScientific) at 37 °C for 4 h. For DNA digest, 1 µL of TURBO DNase Kit was applied to the mixture and incubated for 15 min. The transcribed mRNA was precipitated with lithium chloride suspension and finally resuspended with 60 µL nuclease-free water. The concentration was measured using spectrophotometer NanoDrop2000 and aliquots of mRNA were stored at −80 °C.

Cell-free protein synthesis was performed using the Rabbit Reticulocyte Lysate System (Promega). In total, 4 µg in vitro transcribed mRNA of all K113 ENV was used per reaction. A total of 1 µL of RiboLock RNase inhibitor (ThermoScientific) was used. One reaction without RNA and one reaction without the amino acid L-methionine were used as negative controls. The reactions were set up according to manufacturer’s protocol and incubated in a water bath at 30 °C for 90 min. Subsequently, the digestion of RNA was done using RNaseA (ThermoScientific) at final concentration of 0.2 mg/mL for 5 min. The tubes were set on ice for 10 min to stop the reaction. The translation reaction was stored in SDS sample buffer at −20 °C. For further analysis of protein by SDS-PAGE, an aliquot of 12 µL was mixed with SDS sample buffer and nuclease-free water to 60 µL and boiled for 5 min at 95 °C.

### 4.15. In-Silico Determination of mRNA Secondary Structure

The in-silico prediction of mRNA secondary structure was performed using *The mfold Web Server* (http://unafold.rna.albany.edu/?q=mfold; last accessed data 14.02.2020) [57]. The nucleotide sequences starting at the putative transcription start site and ending at the poly(A) site of the vectors with cloned wildtype or codon optimized HERV *ENV* of K18, K113, and Fc1 were analyzed.

### 4.16. Statistics

For comparison of relative mRNA levels measured with qRT PCR one-way ANOVA followed by Dunnett’s multiple comparison test was performed. Statistical significance was indicated by asterisks (*, *p* < 0.05; ***, *p* < 0.001).

## Figures and Tables

**Table 1:** Sequence coverage and proteolytic peptides identified by LC-MS/MS for expressed HERV proteins and the ER chaperone BiP.

Sample	Protein	Sequence Coverage (%)	Proteolytic Peptides #
Control	K113, Fc1 envelope proteins	n.d.	n.d.
	ER chaperone BiP	20	7
K113	K113 envelope protein	4	3
	ER chaperone BiP	47	31
coK113	K113 envelope protein	20	10
	ER chaperone BiP	48	27
Fc1	Fc1 envelope protein	n.d.	n.d.
	ER chaperone BiP	53	36
coFc1	Fc1 envelope protein	39	25
	ER chaperone BiP	54	38

BiP: binding immunoglobulin protein, n.d.: not detected.

## Supplementary Materials

Supplementary Materials can be found at https://www.mdpi.com/1422-0067/21/21/7855/s1.
