# Supplementary material for: Formation of HERV-K and HERV-Fc1 Envelope Family Members is Suppressed on Transcriptional and Translational Level
Source: Int J Mol Sci. 2020 Oct 23;21(21):7855. doi: 10.3390/ijms21217855 (PMC7660216; doi:10.3390/ijms21217855)
Supplement: Supplementary file 1 [file ijms-21-07855-s001.pdf]

Supplementary Data for

Formation of HERV-K and HERV-Fc1 envelope family members is suppressed on transcriptional and translational level

Victoria Gröger, Lisa Wieland, Marcel Naumann, Ann-Christin Meinecke, Beate Meinhardt, Steffen Rossner, Christian Ihling, Alexander Emmer, Martin S. Staege and Holger Cynis

This file contains:

- Supplementary Table S1-S4
- Supplementary Fig. S1-S11

Supplementary Table S1. List of Primers for cloning HERV sequences. The primer sequences show restriction sites in bold and the respective tag underlined.

**Primer name**	**Sequence 5'-3'**	**Restriction site**
Fc1_mut_C705T_for<br>*Fc1_mut_C705T_rev*	CCTAGTCCGCCACGGGGCTCGCCTTG<br>CAAGGCGAGCCCCGTGGCGGACTAGG	
Fc1_mut_A438T_for<br>*Fc1_ mut_A438T_rev*	CCTAGTCCGCCACGGGGCTCGCCTTG<br>CAAGGCGAGCCCCGTGGCGGACTAGG	
pcDNA_Fc1-Flag_for;<br>*pcDNA_Fc1-EGFP_for*	TATA**GGATCC**ATGGGCAGACCTTCCCCAC	*Bam*HI
pcDNA_Fc1-Flag_rev;<br>*pcDNA_coFc1-Flag_rev*	TATA**GCGGCCGC**TCACTTGTCGTCATCGTCTT<br>TGTAGTCTCTGGCTGCTTCCTGCTG	*Not*I
pcDNA_Fc1-EGFP_rev	TATA**GCGGCCGC**TTTCTGGCTGCTTCCTGCTG	*Not*I
pcDNA_coFc1-Flag_for	TATA**GGATCC**ATGGCCCGCCCTTCAC	*Bam*HI
pcDNA_K18-Flag_for	TATA**AAGCTT**ATGGTAACACCAGTCACATG	*Hind*III
pcDNA_K18-Flag_rev	TATA**GCGGCCGC**TCACTTGTCGTCATCGTCTT<br>TGTAGTCTGGCCCGTTCTCGATGTC	*Not*I
pcDNA_coK18-Flag_for	ATAT**AAGCTT**GCCGCCACCATGGTGACACCT<br>GTGACTTG	*Hind*III
pcDNA_coK113-Flag_for;<br>*pcDNA_coK18*-Flag_for*	ATGCATCTAGAT**AAGCTT**GCCGCCACCATG	*Hind*III
pcDNA_coK113-Flag_rev	CGAT**GCGGCCGC**TTACTTATCGTCATC	*Not*I
pcDNA_coK18-Flag_rev;*<br>*pcDNA_coK18-Flag_rev*	CGAT**GCGGCCGC**TTATTTATCGTCATC	*Not*I

Supplementary Table S2. Antibodies used for Western Blot analysis

Name	Type	Organism	Dilution	Manufacturer
anti-DYKDDDDK (FLAG) tag	primary	rabbit	1:1000	Cell signaling, Cambridge, Great Britain
anti-HERV-K TM HERM1811-5	primary	mouse	1:1000	Austral Biologicals, San Ramon, CA, USA
Anti-HERV-K SU HERM1821-5	primary	mouse	1:1000	Austral Biologicals, San Ramon, CA, USA
anti-β-Aktin	primary	rabbit	1:1000	Cell signaling, Cambridge, Great Britain
anti-mouse IgG-HRP	secondary	horse	1:2000	Cell signaling, Cambridge, Great Britain
anti-rabbit IgG-HRP	secondary	goat	1:2000	Cell signaling, Cambridge, Great Britain

Supplementary Table S3. Antibodies used for immunofluorescence and FACS analysis

**Antibody**	**Dilution**	**Manufacturer**
anti-DYKDDDDK (FLAG) tag	1:800	Cell signaling, Cambridge, Great Britain
anti-HERV-K TM HERM1811-5	1:500	Austral Biologicals, San Ramon, CA, USA
anti-calnexin	1:50	Cell signaling, Cambridge, Great Britain
anti-golgin-97	1:100	Cell signaling, Cambridge, Great Britain
anti-GP73	1:50	Santa Cruz, Heidelberg, Germany
anti-tubulin-α	1:250	BioLegend, San Diego, USA
Cy^{TM}2 anti-rabbit IgG	1:200	Dianova, Hamburg, Germany
Cy^{TM}2 anti-mouse IgG	1:200	Dianova, Hamburg, Germany
Cy^{TM}3 anti-rabbit IgG	1:400	Dianova, Hamburg, Germany
Cy^{TM}3 anti-mouse IgG	1:200	Dianova, Hamburg, Germany

Supplementary Table S4. Primer sequences for qRT PCR

**Target gene**	**Sequence of forward primer 5'-3'**	**Sequence of reverse primer 5'-3'**
DDIT3	TTGCCTTTCTCCTTCGGGAC	TGATTCTTCCTCTTCATTTCC
APOBEC3B	CCATCCTCTATGGTCGGAGC	GAGGCTTGAAATACACCTGGC
APOBEC3G	GCATCGTGACCAGGAGTATGA	GTCAGGGTAACCTTCGGGT
MOV10	GGGCCAGTGTTTCGAGAGTTT	TCTTGGTGACGTAGGCCAGA
HSPA5	GAACGTCTGATTGGCGATGC	TCAACCACCTTGAACGGCAA
sXBP1	CTGAGTCCGCAGCAGGTG	ATGACTGGGTCCAAGTTGTCC
ATF4	TCCAACAACAGCAAGGAGGAT	TCCAACGTGGTCAGAAGGTC
GAPDH	ACCCAGAAGACTGTGGATGG	TTCTAGACGGCAGGTCAGGT
K113 ENV	CCTTGTGTGCCTGTTTTGTC	ATCTCTCTTGCTTTTCCCCACA
coK113 ENV	TGTCTGCTGCTGGTGTATAGG	ATCTGGTCCCTTTTGCTTTTGC
Neo^R	AGACAATCGGCTGCTCTGAT	AGTGACAACGTCGAGCACAG
HPRT1	ACCAGTCAACAGGGGACATAA	CTTCGTGGGGTCCTTTTCACC

Supplementary Figure S1

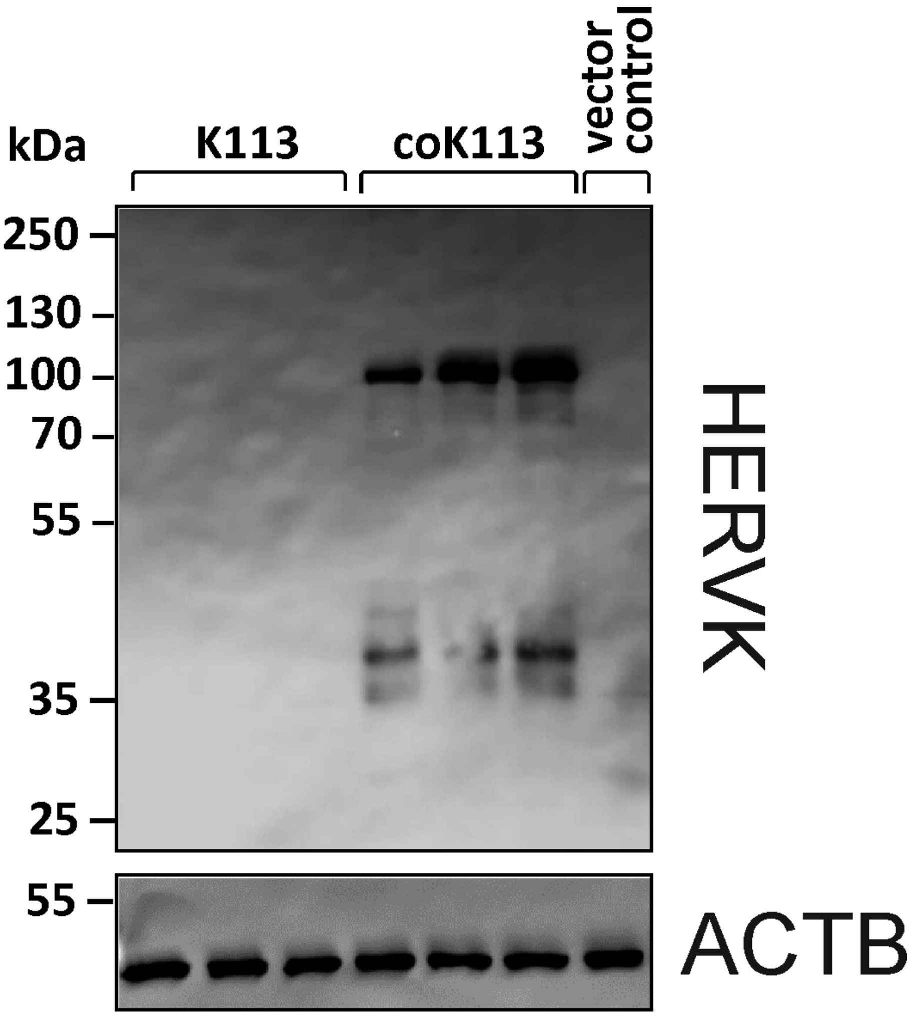

Supplementary Figure S1. Representative Western Blot Image showing the expression of K113 and codon-optimized coK113 in HEK 293 cells in relation to the loading control ACTB (beta-Actin).

Supplementary Figure S2

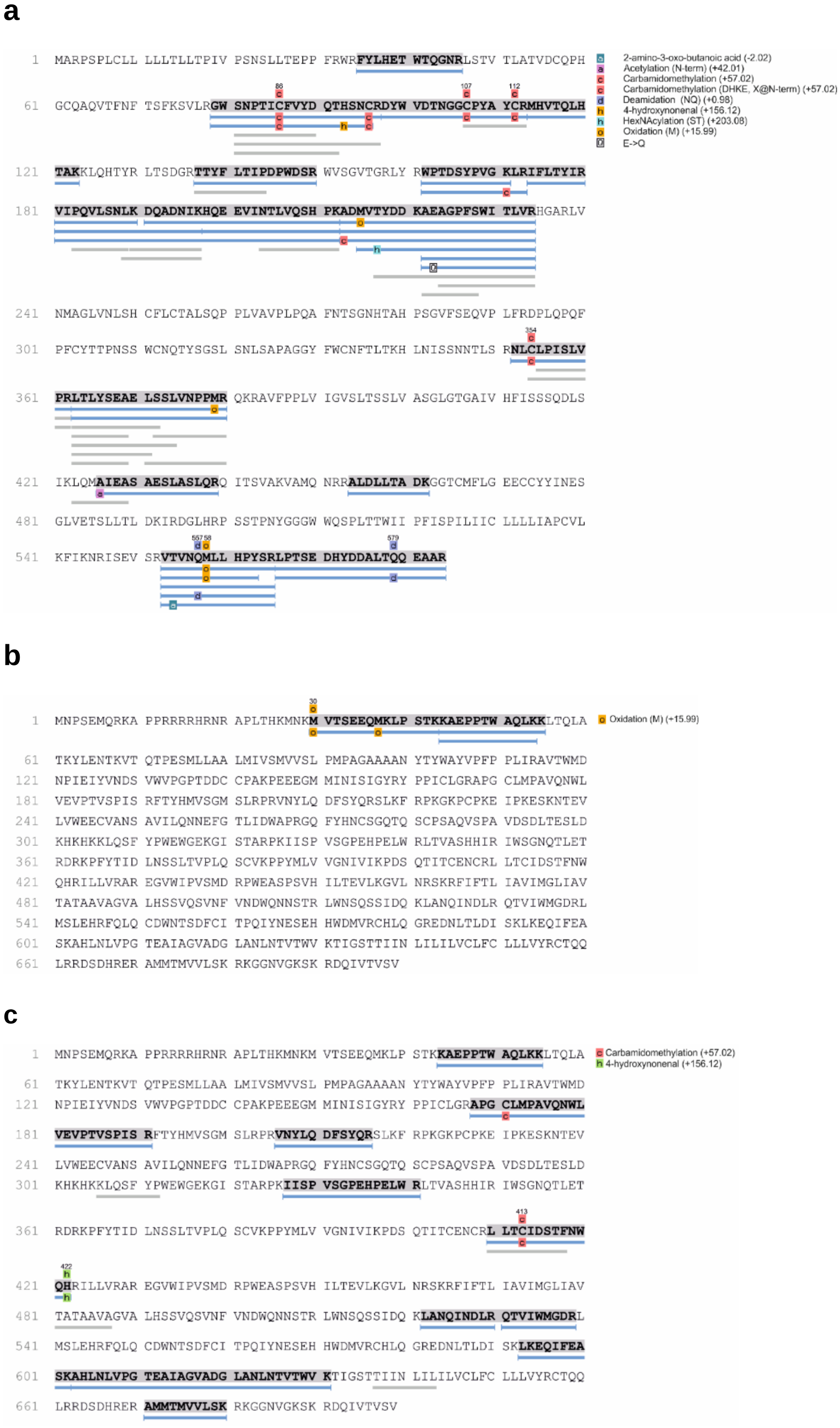

Supplementary Figure S2. Sequence coverage for the envelope proteins of the (a) endogenous retrovirus group Fc1 (UniProt: P60507) identified in HEK293 cells expressing coFc1, (b) endogenous retrovirus group K member 113 (UniProt: Q902F9) identified in HEK293 cells expressing K113 and (c) endogenous retrovirus group K member 113 (UniProt: Q902F9) identified in HEK293 cells expressing coK113.

Supplementary Figure S3

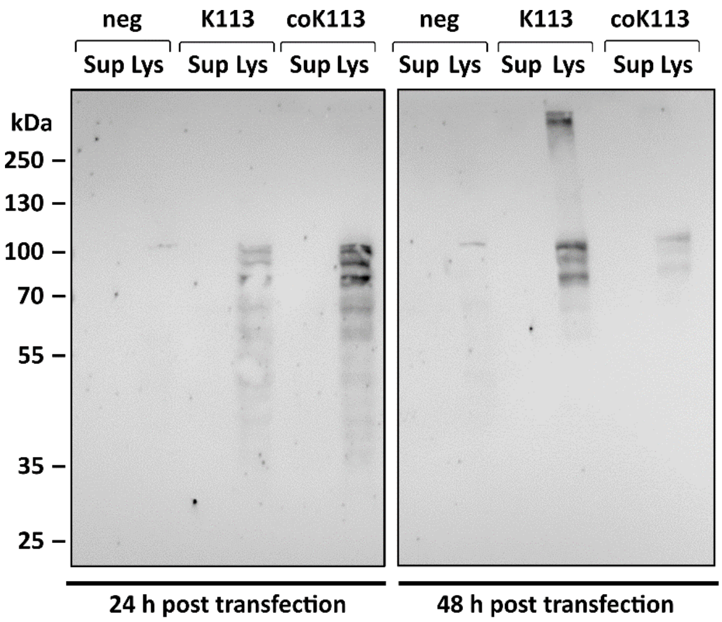

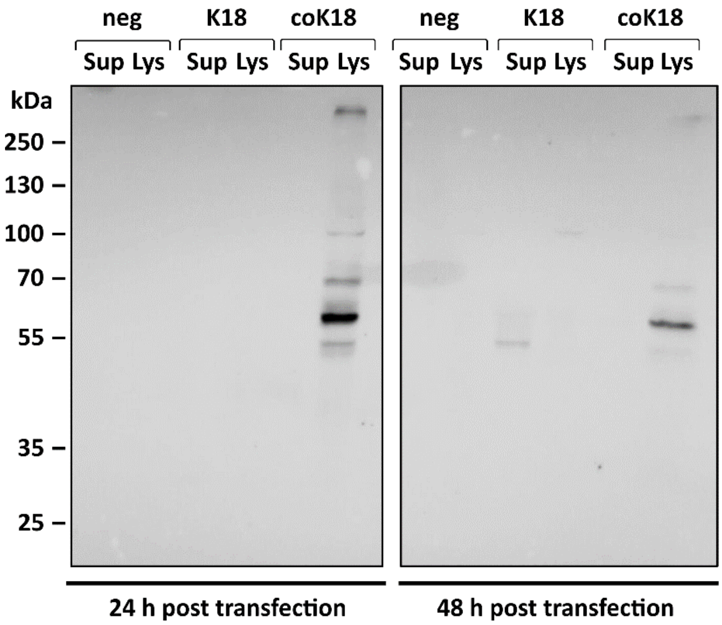

Supplementary Figure S3. Time-dependent expression of WT and codon-optimized variants of K113 and K18. Cell harvest was 24h and 48h post-transfection. For each time-point, 40 µg of cell lysate (Lys) and 20 µl of cell culture supernatant (Sup) were analyzed using a HERV-K antibody specific for the surface unit. HERV expression was compared to empty vector control (neg).

Supplementary Figure S4

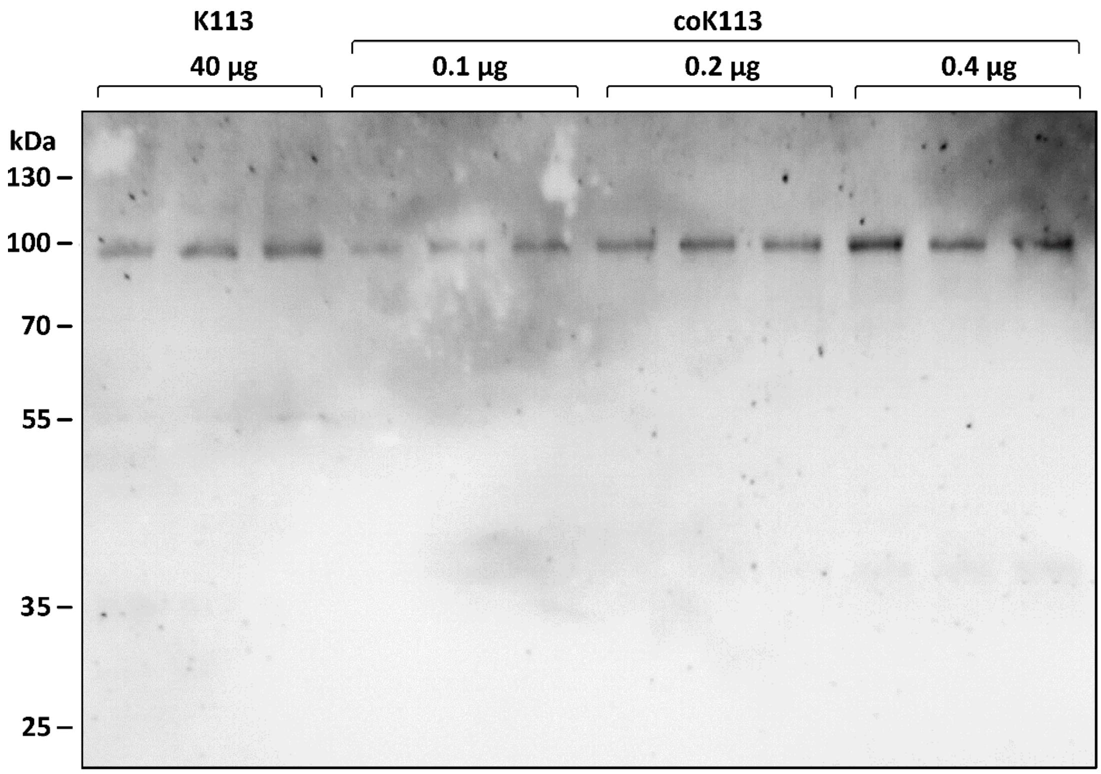

Supplementary Figure S4. Entire image of the blot used for densitometric quantification of expression increase caused by codon-optimization and depicted as Fig. 1d in the main body of the manuscript.

Supplementary Figure S5

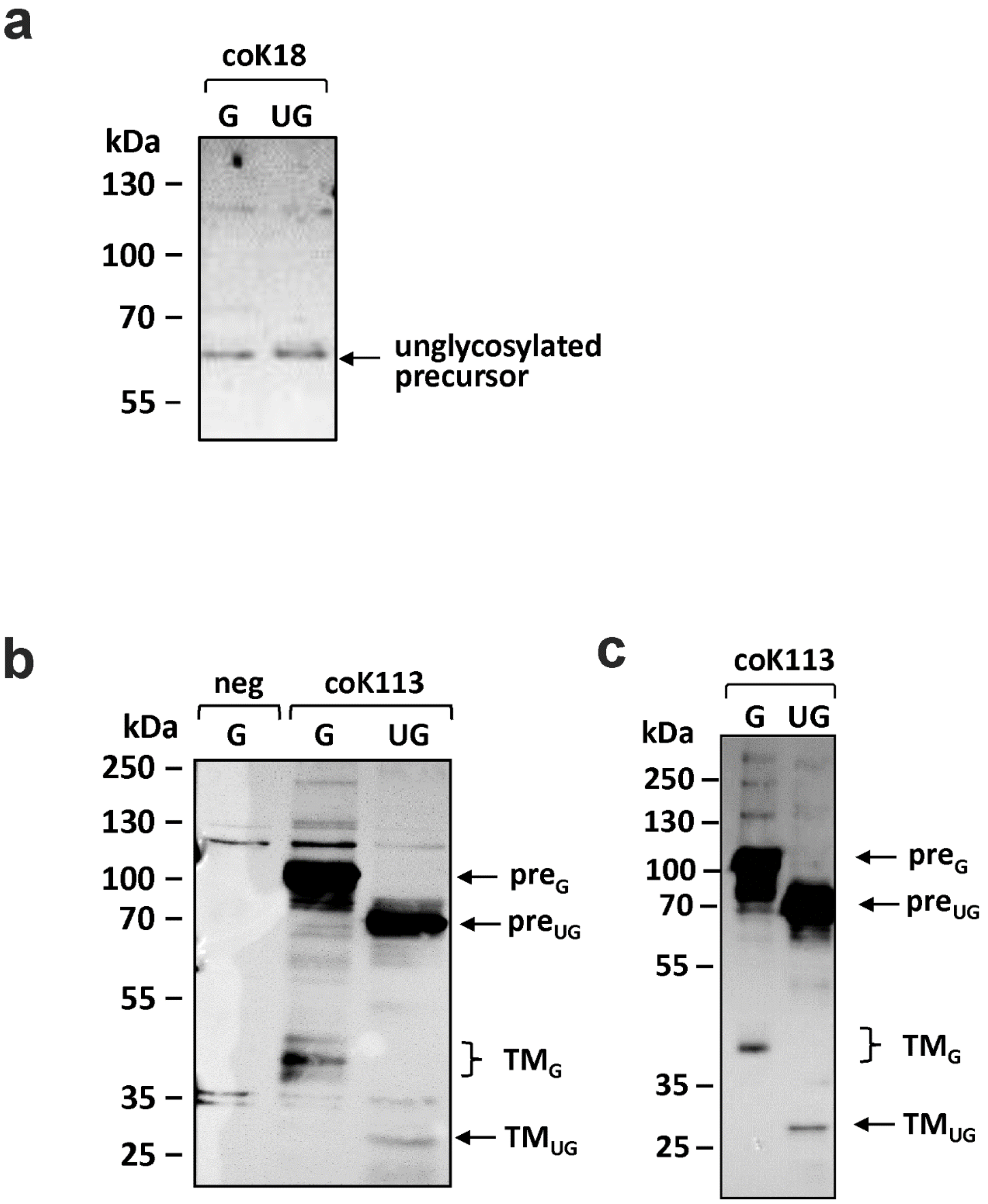

Supplementary Figure S5. (a) No glycosylation of HERV-K18 ENV without signal peptide in transfected HEK293 cells. Cell lysates were analyzed via Western blot 24 h after transient transfection of HEK293 cells with expression plasmids containing codon-optimized (co) sequence of the envelope proteins of HERV-K18 and subsequent treatment with (UG) or without (G) de-glycosylating agent PNGase F. Each lane was loaded with 20 µg total protein. (b) and (c) The cleavage of the precursor (pre) of coK113 into SU and TM is visible, whereby only the precursor and TM were detected by the anti-HERV-K TM HERM1811-5 antibody (two independent experiments). In (b) empty vector-transfected cells served as negative control (neg).

Supplementary Figure S6

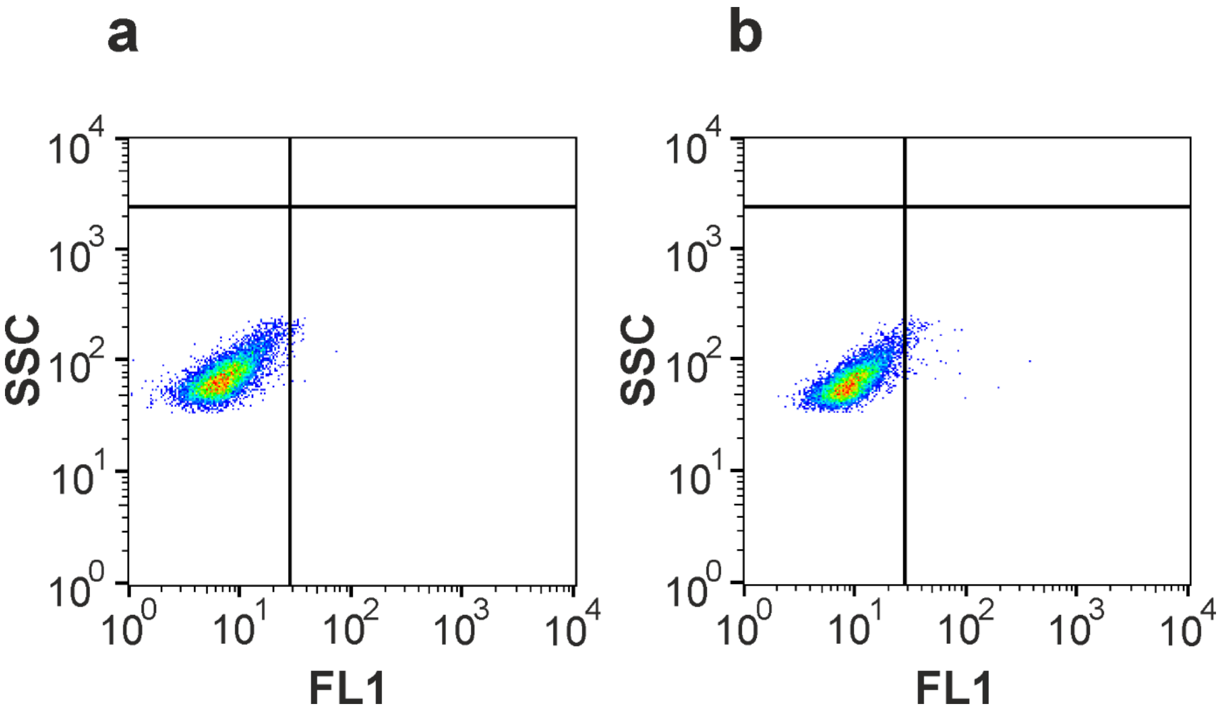

Supplementary Figure S6. Dot plots of HEK293 as determined by FACS analysis. (a) Native HEK293 cells were compared to (b) HEK293 cells incubated with Cy2-labled secondary antibody goat anti-mouse IgG. No unspecific adhesion to HEK293 cells was observed.

Supplementary Figure S7

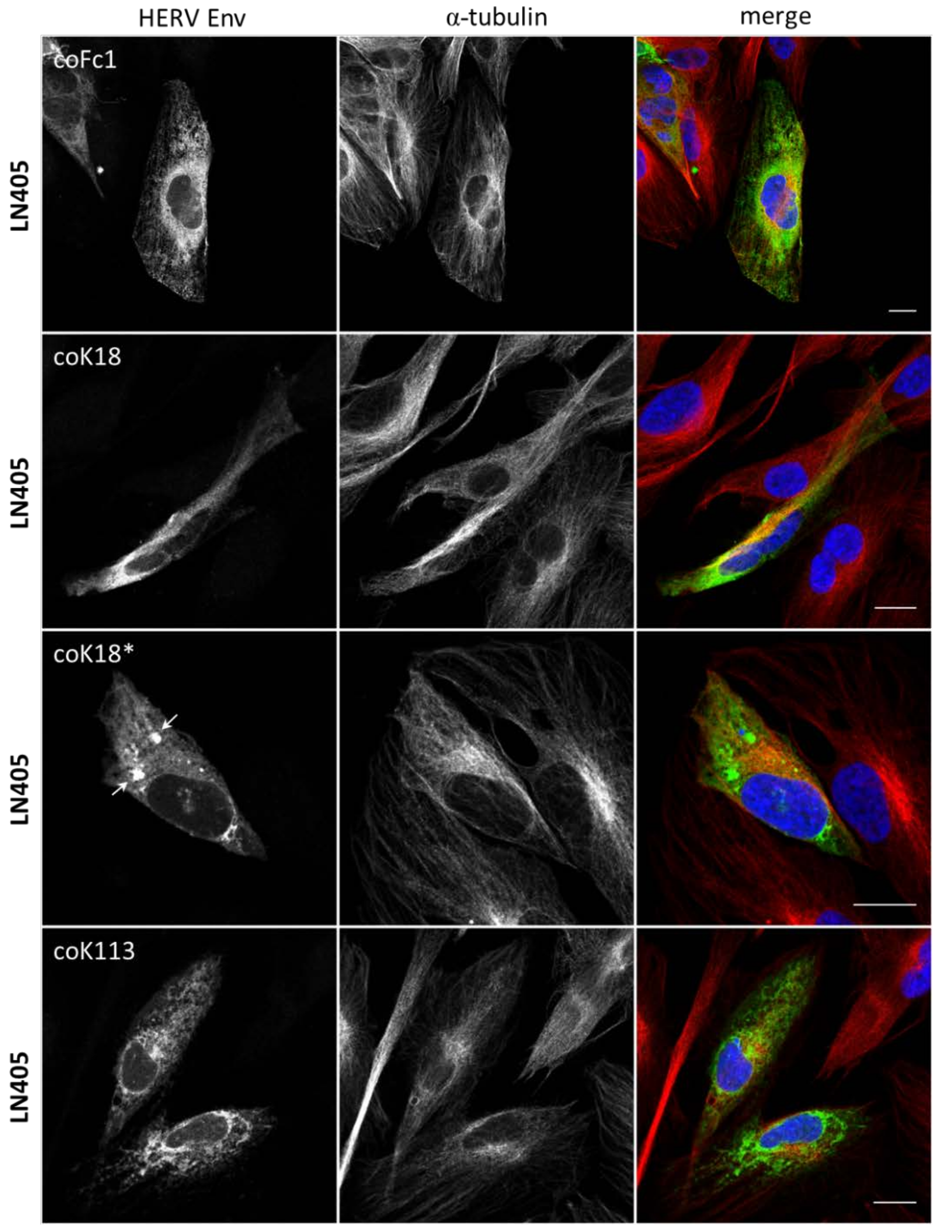

Supplementary Figure S7. Localization of HERV ENV with α-tubulin in transfected LN405 cells. Fluorescence microscopic images showing the subcellular localization of specified HERV ENV and α-tubulin in transiently transfected LN405 cells. The merge image is a z-projection (maximum intensity) of the 3 recorded channels (green: HERV ENV, red: α-tubulin, blue (DAPI): cell nucleus). Pixels with red and green fluorescence appear yellow. The detection of the envelope proteins was performed using anti-HERV-K-TM antibody HERM1811-5 (coK18, coK18*, coK113) or EGFP tag (Fc1). The α-tubulin was visualized by an anti-tubulin-α antibody. Scale bar: 20 µm.

Supplementary Figure S8

a

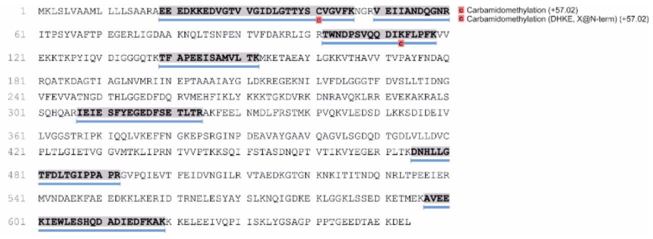

b

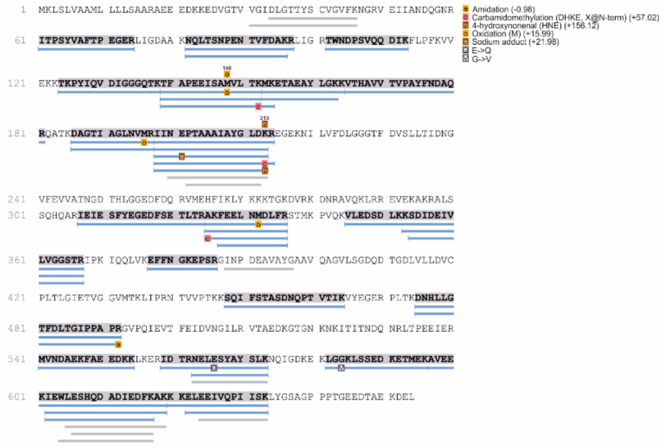

c

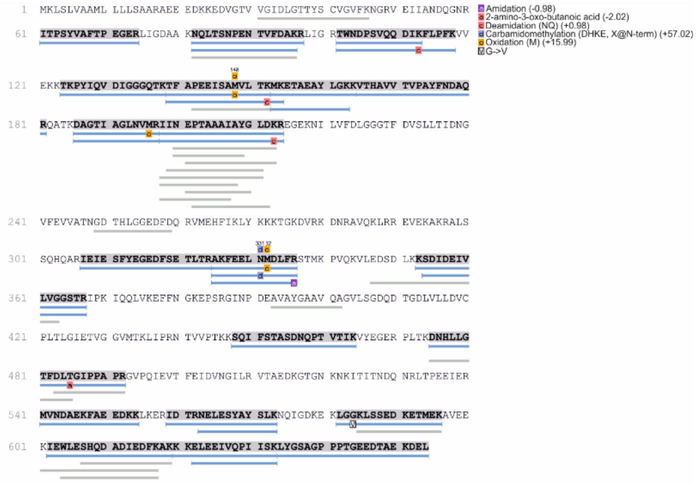

d

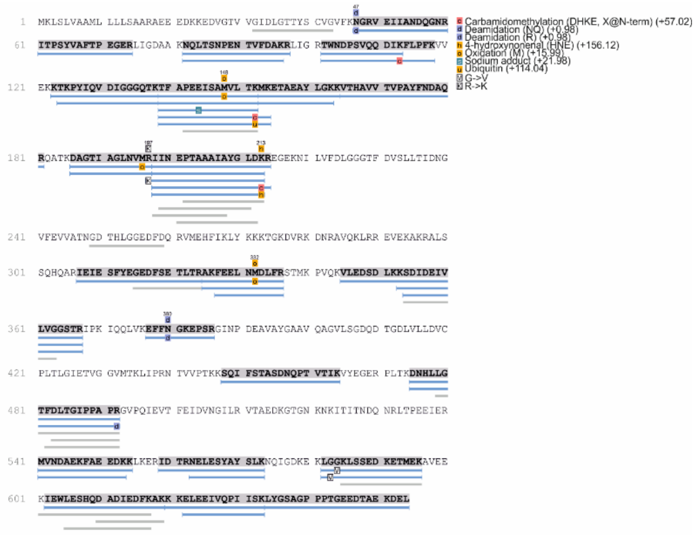

e

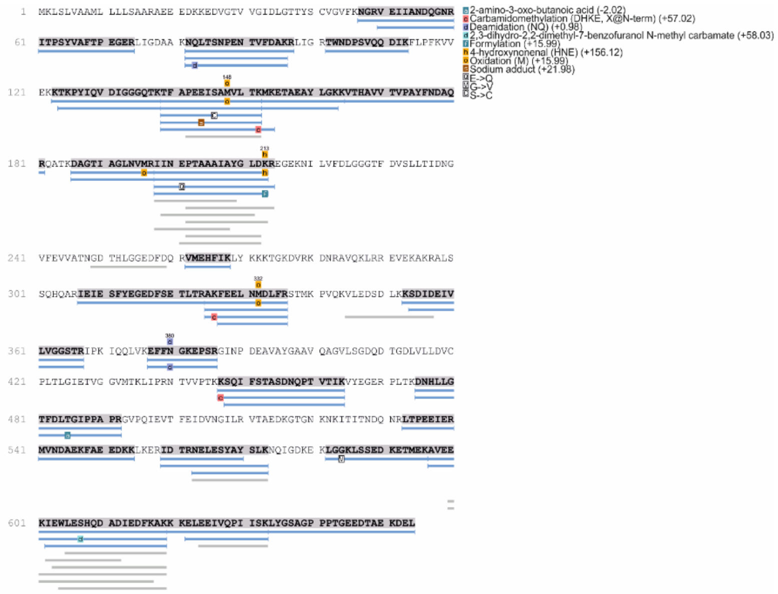

Supplementary Figure S8. Sequence coverage for the endoplasmic reticulum chaperone BiP (UniProt: P11021) identified in (a) the empty vector (pcDNA3.1)-expressing HEK293 cells, (b) K113-expressing HEK293 cells, (c) coK113-expressing HEK293 cells, (d) Fc1-expressing HEK293 cells and (e) coFc1-expressing HEK293 cells.

Supplementary Figure S9

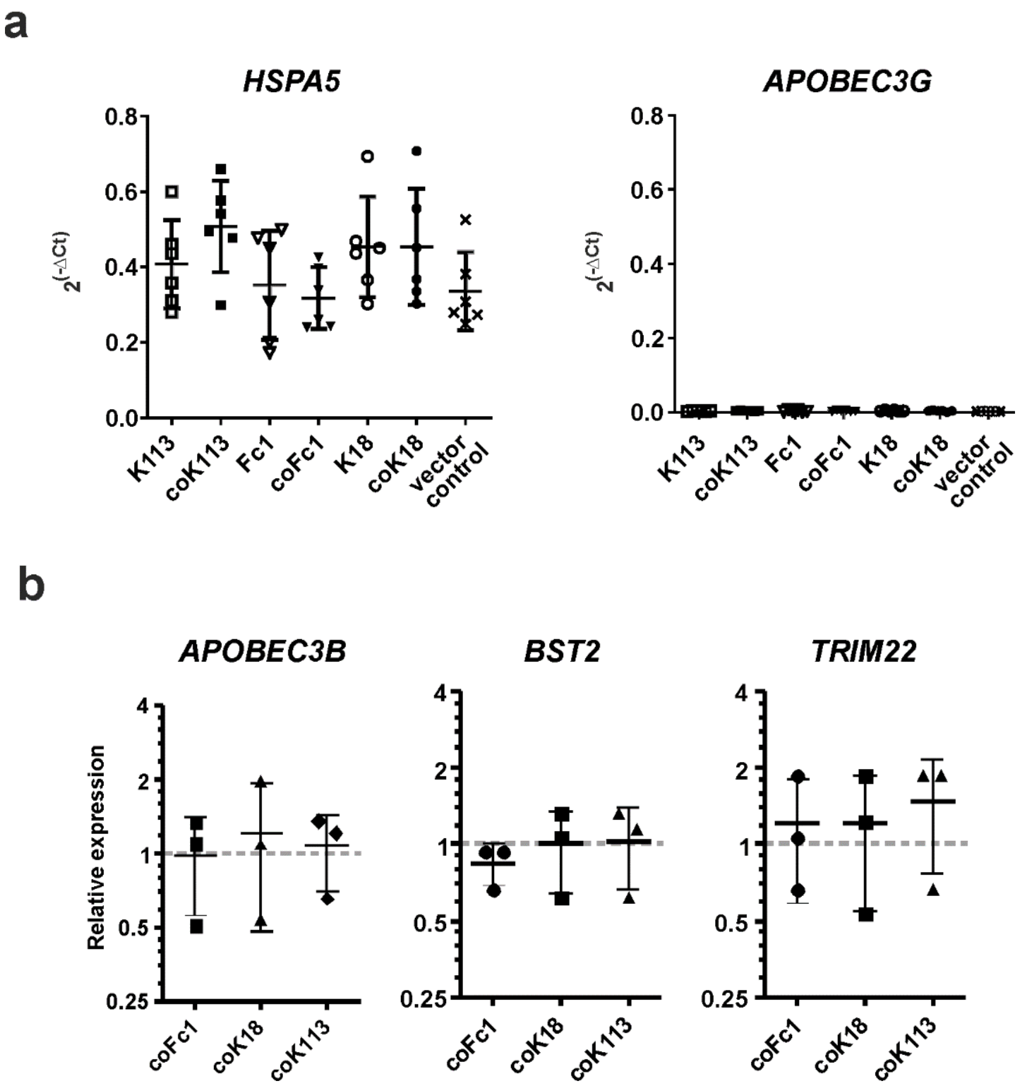

Supplementary Figure S9. (a) Comparison of $2^{(-\Delta Ct)}$ values for *HSPA5* and *APOBEC3G* in HEK293 cells. Expression of *HSPA5* and *APOBEC3G* was analyzed in transfected HEK293 cells by quantitative PCR. The expression was normalized to *GAPDH*. (b) Relative gene expression of additional genes involved in viral defense mechanisms after transfection of codon-optimized HERV envelope protein sequences studied in COS-7 cells (*APOBEC3B*) or HEK293 cells (*BST2*, *TRIM22*). The expression was normalized to *GAPDH* and relative to empty vector-transfected cells (dashed grey line)

Supplementary Figure S10

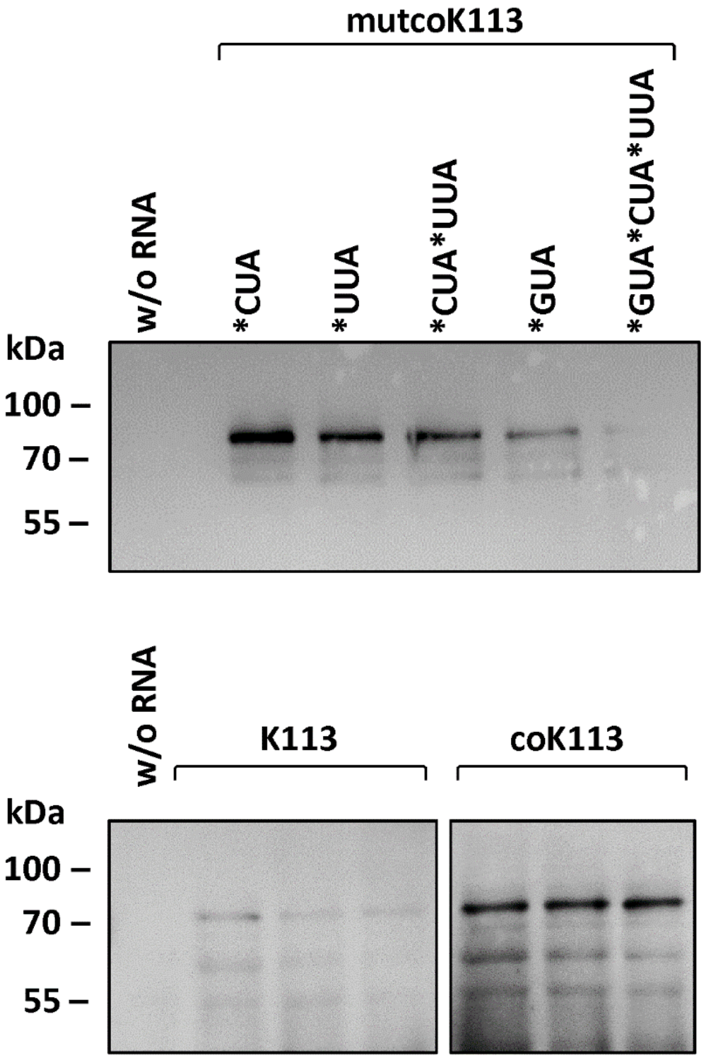

Supplementary Figure S10. Cell-free expression of HERV-K113 envelope variants. 4 µg of transcribed mRNA of K113 envelope sequences were translated into protein using reticulocyte lysate from New Zealand white rabbits including mixture of all components necessary for translation. Three independent experiments of wildtype and codon-optimized and one experiment of mutant variants of codon-optimized K113 envelope protein are shown. A translation reaction without RNA served as negative control. An equal aliquot of all reactions was loaded for Western blotting. The non-glycosylated precursor of K113 ENV was observed at 80 kDa using anti-HERV-K-TM (HERM1811-5) antibody. Densitometric analysis of expression level is shown in Figure 7 of the manuscript.

Supplementary Figure S11

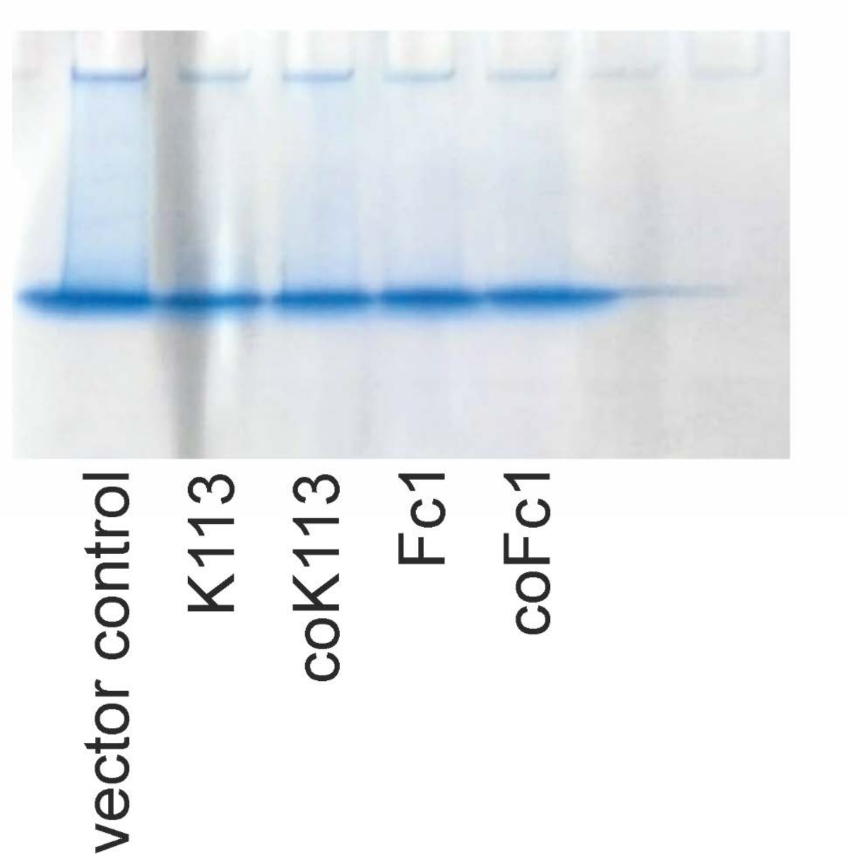

Supplementary Figure S11. 6 % PAA stacking gel used to focus cellular lysates that were subsequently applied to tryptic digestion and LC-MS/MS analysis.
